# The DBC1-HIF-1α-PPAR-γ axis regulates Treg cell differentiation to promote myocardial fibrosis in experimental diabetic cardiomyopathy through the paracrine secretion of Areg

**DOI:** 10.3389/fendo.2026.1780666

**Published:** 2026-04-13

**Authors:** Linhe Lu, Yifei Xu, Changnuan Wen, Qiancong Zhao, Zhihang Li, Jiaqi Liu, Fujie Xu, Jincheng Liu, Jiayou Tang

**Affiliations:** 1 Department of Cardiovascular Surgery, Xijing Hospital, Fourth Military Medical University, Xi’an, China; 2 Department of Physiology and Pathophysiology, National Key Discipline of Cell Biology, Fourth Military Medical University, Xi’an, China; 3 School of Traditional Chinese Medicine, Southern Medical University, Guangzhou, China; 4 Graduate School, Xi’an Medical University, Xi’an, China

**Keywords:** DBC1, diabetic cardiomyopathy, myocardial fibrosis, ST2hiAreghi subgroup, Treg cells

## Abstract

**Background:**

Regulatory T (Treg) cells play crucial roles in myocardial fibrosis, a key pathological feature of diabetic cardiomyopathy (DCM). Deleted in breast cancer 1 (DBC1) has emerged as an inhibitor of the immunosuppressive function of Treg cells in inflammatory states. Here, we studied the subpopulation differentiation and function of Treg cells in the myocardium of DCM and explored the role of DBC1 in Treg cell differentiation.

**Methods:**

DBC1^fl/fl^, DBC1^fl/fl^-Fxop3^Cre^, HIF-1α^fl/fl^-Fxop3^Cre^, PPAR-γ^fl/fl^-Fxop3^Cre^ DCM mouse models were established to evaluate the effects of DBC, HIF-1α, and PPAR-γ on cardiac fibrosis and function. Adoptive transfer therapy was performed to assess the impact of ST2^hi^Areg^hi^ Treg cells. Flow cytometry was used to confirm the role of DBC1 in Treg cell differentiation. Co-culture systems were performed to analyze the effect of ST2^hi^Areg^hi^ Treg cells on fibroblast behaviors.

**Results:**

ST2^+^Treg subsets were increased in the hearts of DCM mice and exhibited increased Areg expression. DBC1 was upregulated in DCM mice and promoted myocardial fibrosis. ST2^hi^Areg^hi^ Treg subsets exhibited increased DBC1 expression and promoted myocardial fibrosis by producing Areg. ST2^hi^Treg subsets promoted the viability, migration, and fibrosis of mouse CFs through Areg paracrine secretion *in vitro*. Furthermore, DBC1 enhanced the differentiation of myocardial Treg cells into the ST2^hi^Areg^hi^ subgroup in DCM through the HIF-1α-PPAR-γ axis.

**Conclusion:**

Our current investigation shows that the DBC1-HIF-1α-PPAR-γ axis promotes the differentiation of myocardial Treg cells into the ST2^hi^Areg^hi^ pro-fibrotic subgroup, which drives maladaptive myocardial fibrosis and cardiac dysfunction via paracrine secretion of Areg in DCM mice.

## Introduction

Diabetic cardiomyopathy (DCM) is a prevalent cardiovascular complication of diabetes mellitus, characterized by structural and functional abnormalities in the myocardium without hypertension or coronary artery disease. Despite extensive research, DCM continues to present a considerable challenge in the comprehensive management of patients with diabetes (1, 2). A key pathological feature of DCM is myocardial fibrosis, which involves excessive accumulation of extracellular matrix components and alterations in cellular architecture. Myocardial fibrosis plays a pivotal role in the deterioration of diastolic function in the left ventricle and results in impaired myocardial compliance and contractility, ultimately leading to heart failure (3). Recently, it is increasingly recognized that dysregulation of immune responses significantly contributes to the development of myocardial fibrosis (4, 5). Chronic inflammation, characterized by the infiltration of immune cells and the release of pro-fibrotic cytokines, enhances the fibrotic remodeling of cardiac tissue (6, 7). Despite the growing understanding of the role of immune dysfunction in DCM, the precise mechanisms through which immune imbalances induce myocardial fibrosis remain inadequately understood.

Regulatory T (Treg) cells, characterized by the expression of the transcription factor Foxp3, play a crucial role in maintaining immune homeostasis and preventing excessive inflammation within tissues. These specialized CD4+ T cells are crucial components of the local immune suppressive environment, functioning through direct cell-cell interactions and the secretion of anti-inflammatory cytokines as well as tissue repair molecules (8). Given their ability to modulate inflammation and promote tissue repair, Treg cells have emerged as a promising area of research for DCM treatment (9). In pathological inflammatory states, however, Treg cells often exhibit instability and altered functional differentiation into various subpopulations (10). Areg plays a key role in tissue repair and has been implicated in enhancing the regenerative properties of Treg cells during inflammation (11, 12). ST2 is known to mediate the biological effects of IL-33, and its expression on Treg cells can influence their functional outcomes in response to pathological stimuli. As an example, in the myocardium of a mouse model of ischemia-reperfusion injury, Treg cells differentiate into a pro-fibrotic functional subset under the regulation of the IL-33/ST2 axis, thereby increasing cardiac collagen content and contributing to the maturation of the infarcted area (13). These studies have focused on Treg cells expressing high levels of ST2 and Areg (ST2^hi^Areg^hi^ subgroup), revealing their potential roles in facilitating fibrosis and remodeling of heart tissues. Nonetheless, studies on the functional stability and subpopulation differentiation of Treg cells in the myocardium of DCM remains scarce.

Deleted in breast cancer 1 (DBC1) is a multifaceted protein that has garnered attention for its role in metabolic syndrome and tissue inflammation (14). Deletion of DBC1 safeguards preadipocytes from cellular senescence and the inflammation associated with senescence (15). It has been identified as a key target that enhances inflammatory responses, thereby contributing to the worsening of various metabolic disorders, including diabetes (16). Importantly, our previous report highlights that DBC1 has emerged as a novel molecular player that modulates the stability and function of Treg cells in inflammatory states (17). Upon activation by inflammatory factors, DBC1 negatively regulates the Treg transcriptional complex by interacting with Foxp3, thereby impairing the immunosuppressive functions of Treg cells (17). However, the specific role and mechanisms of DBC1 in affecting Treg cell subpopulation differentiation in DCM remain to be fully elucidated.

In this study, using a mouse model of DCM, we show that ST2^hi^Areg^hi^ Treg subsets are increased in the hearts of DCM mice, and they contribute to myocardial fibrosis by secreting Areg. Further, we sought to explore whether DBC1 is responsible for Treg cell differentiation into ST2^hi^Areg^hi^ subsets in DCM mice, revealing that DBC1 induces Treg ST2^hi^Areg^hi^ differentiation through the HIF-1α-PPAR-γ axis, a critical regulatory mechanism in Treg cell differentiation (18). Understanding the impact of DBC1 on Treg cell differentiation could offer new insights into the immunological aspects of DCM and potentially lead to novel therapeutic strategies targeting Treg cell-mediated fibrosis in the heart.

## Materials and methods

### Mice and ethics statement

All animal experiments were performed in accordance with the guidelines approved by the Animal Ethics Committee of Xijing Hospital. Wild-type (Control) C57BL/6 mice, HIF-1α^fl/fl^ mice, PPAR-γ^fl/fl^ mice, and Foxp3^Cre^ C57BL/6 mice (with Cre recombinase knocked into the Foxp3 locus to achieve Treg-specific gene editing) were purchased from Vital River Laboratories (Beijing, China). DBC1 floxed mice (DBC1^fl/fl^, with loxP sites flanking exons 2–4 of the DBC1 gene) on the C57BL/6 background were generated by our laboratory using CRISPR/Cas9-mediated gene editing. Treg-specific DBC1 conditional knockout mice (DBC-CKO, DBC1^fl/fl^-Foxp3^Cre^), Treg-specific HIF-1α conditional knockout mice (HIF-1α-CKO, HIF-1α^fl/fl^-Foxp3^Cre^), and Treg-specific PPAR-γ conditional knockout mice (PPAR-γ-CKO, PPAR-γ^fl/fl^-Foxp3^Cre^) were generated by crossing DBC1^fl/fl^, HIF-1α^fl/fl^, or PPAR-γ^fl/fl^ mice with Foxp3^Cre^ mice, respectively. Age- and sex-matched DBC1fl/fl littermates without Cre recombinase were used as the control for DBC-CKO mice, following the standard reporting guidelines for conditional knockout mouse models (19). Sex-matched 8-week-old male mice were used in all animal experiments, and all mice were housed in a SPF environment at 22 ± 2 °C with a 12 h light/dark cycle and free access to water and food.

Genotyping and knockout efficiency validationGenomic DNA was extracted from mouse tail tissues using a TIANamp Genomic DNA Kit (Tiangen, Beijing, China), and PCR was performed to identify the genotype of all mice. To validate the Treg-specific knockout efficiency of DBC1, CD4^+^CD25^+^Foxp3^+^ Treg cells and non-Treg cells were flow-sorted from mouse heart tissues. Total protein were extracted from sorted cells, and immunoblot assay were performed to detect the protein level of DBC1, respectively, to confirm the effective and specific deletion of DBC1 in Treg cells.

### Generation of DCM mouse models and animal experiments

Before the establishment of DCM models, the type 2 diabetes (T2DM) mice were generated by high-fat diet (HFD) intake. Briefly, Control, DBC1^fl/fl^, DBC1-CKO, HIF-1α-CKO, and PPAR-γ-CKO C57BL/6 male mice were fed a 45% HFD for 12 weeks prior to overnight fasting. Then, mice were subjected to intraperitoneal injection of streptozotoxin (STZ, 100 mg/kg, Sigma-Aldrich, Saint-Aubin, France) dissolved in citrate buffer (pH=4.5) on the ice. Five days after STZ injection, blood was collected by cutting the tail, and fasting blood glucose was measured using a glucose meter (Johnson & Johnson, New Brunswick, NJ, USA), with a successful T2DM model indicated by a blood glucose level ≥ 250 mg/dL. The control group of mice was fed a normal diet until the corresponding time point and received a simple intraperitoneal injection of 0.1 mM citrate buffer at a dose of 100 mg/kg. In the DBC1-CKO +Ad.DBC1 group, after successful establishment of the T2DM model, the diabetic mice were subjected to injection of DBC1 adenovirus (Ad.DBC1) into three points in the left ventricle (2 × 10^10^ TU/mouse). In the DCM+IL-6/IL-10 groups, the diabetic mice were subjected to intraperitoneal injection of recombinant mouse IL-6 or IL-10 protein (50 µg/kg, Proteintech, Wuhan, China) dissolved in PBS. Four to eight weeks after the successful establishment of the T2DM model, serum and heart samples were collected for subsequent analyses. These animal experiments included a total of nine groups: 1) Control (sham), 2) DCM (Model), 3) DBC1^fl/fl^ (DBC1^fl/fl^-DCM), 4) DBC1-CKO (DBC1-CKO-DCM), 5) DBC1-CKO+ Ad.DBC1, 6) DCM+IL-6, 7) DCM+IL-10, 8) HIF-1α-CKO, and 9) PPAR-γ-CKO.

### Isolation and culture of Treg cells

Treg cells and ST2^hi^Treg cells were isolated from mouse hearts of sham mice, DCM mice, and DBC1-CKO mice. For the isolation of Treg cells, the hearts were quickly excised and rinsed with PBS before being placed in a culture dish. The tissues were ground and filtered, followed by processing using a lymphocyte separation column (Cedarlane, Burlington, ON, Canada). CD4+ T cells were firstly isolated using CD4-specific MACS beads (Miltenyi Biotec, Bergisch Gladbach, Germany). After that, CD4+CD25+ Treg cells were further sorted using CD25 magnetic beads (Invitrogen, Bleiswijk, the Netherlands) via flow cytometry. The sorted cells were then directly placed in a 37 °C incubator for cell culture. For the isolation of ST2^+^Treg cells, isolated Treg cells were incubated with an APC labeled antibody to ST2 (#RMST2-33, Invitrogen) for 45 min at 4 °C. ST2^+^Treg cells were further sorted by flow cytometry. For cell cultivation (37 °C, 5% CO_2_), RPMI-1640 (Gibco, Schwerte, Germany) was used and enriched with 10% FBS (Gibco), 2 mM L-glutamine (Sigma-Aldrich), 1% penicillin/streptomycin (Beyotime, Shanghai, China), and 10 U/mL recombinant IL-2 (Proteintech).

### Adoptive transfer of ST2^hi^Treg cells

The adoptive transfer experiment included 4 groups, with 6 mice in each group: 1) DBC1^fl/fl^ group: Recipient DCM mice received adoptive transfer of ST2^hi^ Treg cells from DBC1^fl/fl^ mice + myocardial injection of empty control adenovirus (Ad-NC); 2) DBC1^fl/fl^-Treg group: Recipient DCM mice received adoptive transfer of ST2^hi^ Treg cells from DBC1^fl/fl^ DCM mice + Ad-NC injection; 3) CKO-Treg group: Recipient DCM mice received adoptive transfer of ST2^hi^ Treg cells from DBC1-CKO DCM mice + Ad-NC injection; 4) CKO-Treg+Ad.Areg group: Recipient DCM mice received adoptive transfer of ST2^hi^ Treg cells from DBC1-CKO DCM mice + myocardial injection of Areg-encoding adenovirus (Ad.Areg). ST2^hi^ Treg cells were isolated from the corresponding donor mice as described above. After successful establishment of the DCM model, ST2^hi^ Treg cells (1 × 10^6^ cells/mouse) were adoptively transferred into recipient DCM mice via a single tail vein injection. Immediately after cell transfer, mice received a single myocardial spot injection of adenovirus at three sites in the left ventricle (total injection volume: 20 μL/mouse): the CKO-Treg+Ad.Areg group received replication-deficient Ad.Areg (1×10^9^ PFU/mouse, Hanbio, Shanghai, China), while the other three groups received the same titer and volume of matched replication-deficient empty adenovirus (Ad-NC, negative control).

### Evaluation of the HW/BW ratio

Using an electronic balance (Ohaus, Shanghai, China), the body weight (BW) and heart weight (HW) of the mice were gauged and the HW/BW ratio was determined.

### H&E, Masson’s trichrome, and Sirius red staining methods

After being fixed in 4% paraformaldehyde, heart samples were embedded in paraffin and subsequently sectioned into 4 μm thick slices. H&E staining was carried out using a commercial Kit from Beyotime, followed by quantification of cross-sectional area of left ventricular myocardium. Masson staining on the 4-μm slices was performed to examine the changes of collagen fibers using a commercial staining Kit (Servicebio, Wuhan, China). A Modified Sirius Red Stain Kit (G-clone, Beijing, China) was applied for Sirius red staining to evaluate collagen fibers in heart sections. Microscopic images were captured using a CKX53 inverted microscope (Olympus, Tokyo, Japan), and data analysis was done by ImageJ (NIH, Bethesda, MD, USA). For histological quantification, 3–5 non-overlapping heart sections per mouse (N = 3-5) were analyzed at 100 μm intervals. Data from 5 mice per group (n = 5) were pooled for statistical analysis.

### Immunofluorescence and immunohistochemistry

Under standard methods as described (20), probing for Foxp3, collagen I, or collagen III in heart sections was conducted. For Foxp3 probing by immunofluorescence, the heart sections were subjected to incubation with an anti-Foxp3 antibody (#22228-1-AP, Proteintech, 1:600) and Alexa Fluor 488-labeled secondary antibody (#ab150077, Abcam, Cambridge, UK, 1:800). Following DAPI staining for cell nucleus, fluorescent images were obtained by BX63 microscope (Olympus). For collagen I or collagen III probing by immunohistochemistry, we incubated the heart sections with anti-collagen I (#67288-1-Ig, Proteintech, 1:5000) or anti-collagen III (#PA5-99160, Invitrogen, 1:150) antibody. After application with HRP secondary antibody, signals were developed by DAB methods (Beyotime).

### Echocardiography

At the end of the animal experiment, the Vevo 2100 Imaging System (VisualSonics, Toronto, ON, Canada) was applied for M-mode transthoracic echocardiography. After being anesthetized, the mice were positioned on a heating platform maintained at 37 °C. By using a 30 MHz linear transducer, left ventricular fractional shortening (LVFS), left ventricular ejection fraction (LVEF), and maximum rise rate and maximum fall rate of left chamber pressure (± dP/dt) were scored automatically.

### ELISA for serum IL-6, TNF-α, IL-10, and IFN-γ levels and cellular Areg content

For these assays, in accordance with the suggestions provided by Enzyme-linked Biotechnology (Shanghai, China), we used a commercially available Mouse IL-6 ELISA Kit, a Mouse TNF-α ELISA Kit, a Mouse IL-10 ELISA Kit, a Mouse IFN-γ ELISA Kit, and a Mouse Areg ELISA Kit. The PowerWave340™ reader (BioTek, Bad Friedrichshall, Germany) was used for absorbance measurement.

### Flow cytometry

Flow cytometry analysis and fluorescence-activated cell sorting (FACS) were performed using the CytoFLEX LX platform (Beckman Coulter, Fullerton, CA, USA). All staining procedures followed the standardized protocol for Treg cell intracellular and surface marker detection.

Cell preparation and viability staining: Freshly harvested cardiac single-cell suspensions or isolated Treg cells were first stained with the Live/Dead Fixable Aqua Dead Cell Stain Kit (Invitrogen) for 15 min at 4 °C to exclude dead cells.

Surface marker staining: After washing, cells were incubated with surface marker antibodies for 30 min at 4 °C in the dark: Brilliant Violet 510-anti-CD45 (#103138, BioLegend), APC-Cy7-anti-CD3 (#100222, BioLegend), FITC-anti-CD4 (#100406, BioLegend), and APC-anti-ST2 (#17-9335-82, Invitrogen, 1:100).

Intracellular fixation and permeabilization: For intracellular staining of Areg and Foxp3, cells were fixed and permeabilized using the Foxp3/Transcription Factor Staining Buffer Set (Invitrogen) according to the manufacturer’s instructions. A PE-Cy7-conjugated monoclonal anti-Areg antibody (#25-5370-41, Invitrogen, 1:100) was used for direct detection. Isotype controls and fluorescence-minus-one (FMO) controls were included to set gating thresholds. No secondary antibodies were used, as all intracellular markers (Foxp3, Areg) were detected with directly conjugated primary antibodies. Isotype control antibodies and FMO controls were included in each independent experiment to set the positive gating thresholds.

Gating strategy: The sequential gating was performed as follows: 1) FSC-A/SSC-A to gate the lymphocyte population; 2) FSC-H/FSC-A to exclude doublets and select singlets; 3) Live/Dead staining to select viable cells; 4) CD45^+^CD3^+^ to gate immune T cells; 5) CD4^+^ to gate CD4^+^ T cells; 6) Foxp3^+^ to gate Treg cells; 7) ST2 and Areg expression analysis within the Foxp3^+^ Treg population.

Definition of ST2^hi^ and Areg^hi^ populations: The positive threshold for ST2 and Areg was set based on FMO controls, with fluorescence intensity above the threshold defined as positive. The ST2^hi^ and Areg^hi^ subsets were defined as the top 20% of Foxp3^+^ Treg cells with the highest fluorescence intensity of ST2 and Areg, respectively, consistent with previous published criteria for Treg functional subset definition (21).

### Constructs and transfection of Treg cells

For *in vitro* transfection experiments, we procured pLV3-CMV-Ccar2(mouse)-3×FLAG-CopGFP-Puro (OE-DBC1) and pCMV-Hif1a(mouse)-3×FLAG-Neo (pc-HIF-1α) from Miaoling Biology (Wuhan, China), Silencer^®^ Select siRNA targeting mouse DBC1 (si-DBC1) from Thermo Fisher Scientific (Milan, Italy), and PPAR-γ Mouse Pre-designed siRNA set A (si-PPAR-γ) from MCE (Shanghai, China). Using Lipofectamine 3000 as the transfection reagent, transient introduction of OE-DBC1, si-DBC1, si-DBC1+pc-HIF-1α, or si-DBC1+pc-HIF-1α+si-PPAR-γ into Treg cells was performed as suggested by the vendor (Invitrogen). Subsequent analyses were conducted at 48 h post-transfection.

### Isolation of mouse cardiac fibroblasts

In accordance with a previously reported method (22), mouse CFs were isolated from C57BL/6 male mice. Briefly, after being minced and washed, heart tissues were pre-digested in an enzyme solution (0.1 mg/mL pancreatin and 100 U/mL collagenase II) at 37 °C for 15 min, followed by 6×20-min digestion rounds. After that, the supernatants were harvested, added with FBS, and subjected to centrifugalization (1,000 rpm, 5 min). The pellet was resuspended in 10% FBS DMEM and filtered through a 100 μm cell strainer. Isolated CFs were maintained at 37 °C in 5% CO_2_ and used for experiments at passages 2-4.

### Co-culture systems

To evaluate the impact of ST2^hi^Treg cells on CF behaviors, mouse CFs were co-cultured with ST2^hi^Treg cells isolated from the cardiac tissues of WT DCM mice (WT_Treg_) or DBC1-CKO DCM mice (CKO-Treg). Co-culture systems were established using 24-well Transwell inserts (Corning, Lindfield, NSW, Australia): CFs were seeded in the upper inserts with 10% FBS DMEM, and ST2^hi^Treg cells were added to the bottom compartment with 10% FBS RPMI-1640. For intervention experiments, the culture medium in the bottom compartment was supplemented with anti-Areg neutralizing antibody (10 μg/mL, #PA5-16621, Invitrogen), recombinant mouse Areg protein (rmAreg, 50 ng/mL, #315-36-50UG, Invitrogen), or an equal volume of PBS (vehicle control for rmAreg), following the standardized intervention protocol for Areg *in vitro*. After 24–36 h of co-culture, cell viability, migratory ability, and collagen expression of CFs were evaluated.

The co-culture system was established at a Treg: CF ratio of 1:5, with 1 × 10^5^ ST2^hi^Treg cells seeded in the lower chamber and 5 × 10^5^ mouse cardiac fibroblasts (CFs) seeded in the upper Transwell insert (0.4 μm pore size). The anti-Areg neutralizing antibody was used at a final concentration of 10 μg/mL, and recombinant mouse Areg (rmAreg) was applied at 50 ng/mL, based on established protocols in the literature (23, 24).

### Cell viability and migration assays

For viability analysis, co-cultured CFs were seeded in a 96-well plate and maintained for 6 h at 37 °C, followed by CCK-8 assay as suggested by the supplier (Beyotime). For transwell migration assay, the 24-Tranwell inserts in the co-culture system were stained with 0.5% crystal violet, and the migrated cells were quantified under the CKX53 microscope. For wound healing assay, co-cultured CFs were seeded in 6-well plates and grown to ~100% confluent, followed by the generation of a vertical wound. After 24 h, the healing ratio (%) was determined.

### Quantitative PCR

For RNA preparation from mouse hearts or cultured Treg cells, a BeyoMag™ RNA Kit was applied as suggested by the vendor (Beyotime). According to the suppliers’ recommendations, a PrimeScript RT Reagent Kit (TaKaRa, Dalian, China) was used for cDNA synthesis, and quantitative PCR was conducted using SYBR Green Mix (Servicebio) and designed primers. Mouse β-actin served as the reference gene, and gene relative expression was determined using the formula 2^-ΔΔCt^.

### Immunoblot assay

Under the application of a Total Protein Extraction Kit from Abcam, we prepared protein samples from mouse hearts, cultured Treg cells or co-cultured CFs. As described elsewhere (25), immunoblotting was carried out using anti-DBC1 (#PA5-54113, Invitrogen, 1:300), anti-collagen I (#67288-1-Ig, Proteintech, 1:10000), anti-collagen III (#PA5-99160, Invitrogen, 1:1000), anti-MMP-3 (#ab52915, Abcam, 1:8000), anti-HIF-1α (#3716, Cell Signaling Technology, Danvers, MA, USA, 1:1000), anti-PPAR-γ (#2435, Cell Signaling Technology, 1:1000), or anti-GAPDH (#ab9485, Abcam, 1:2500) antibody. Signal development was achieved using ECL procedure (Merck Millipore, Darmstadt, Germany), and blot quantification was done by ImageJ.

### Measurement of ROS, SOD, MDA, and GSH-Px

For these detections, we harvested mouse hearts and employed a commercial ROS Assay Kit (Beyotime), a SOD Assay Kit (Beyotime), a MDA Assay kit (Abcam), and a GSH-Px Assay kit (Enzyme-linked Biotechnology), following the accompanying instructions.

### Statistical analysis

All results are expressed as mean ± standard deviation (SD). All statistical analyses were performed using GraphPad Prism 10.0 software (GraphPad Software, San Diego, CA, USA). Data pre-assessment: For all datasets, the normality of data distribution was first tested using the Shapiro-Wilk test, and homogeneity of variances was verified using Levene’s test. Parametric tests were applied for datasets meeting both normality and equal variance assumptions; non-parametric tests (Mann-Whitney U test for two groups, Kruskal-Wallis test with Dunn’s *post-hoc* test for ≥3 groups) were used for datasets that failed the normality or variance homogeneity test. Specific statistical tests for each experimental design: Comparisons between two independent groups were performed using an unpaired two-tailed Student’s t-test. Comparisons among three or more independent groups with a single variable were analyzed using one-way ANOVA, followed by the Bonferroni *post-hoc* test for multiple pairwise comparisons to control for type I error. Comparisons among groups with two independent variables were analyzed using two-way ANOVA, followed by the Bonferroni *post-hoc* test for multiple pairwise comparisons. Definition of replicates: For all experiments, n represents the number of independent biological replicates (i.e., individual mice, independent cell culture preparations, or distinct experimental batches), not technical replicates. Technical replicates were only used for signal averaging in qPCR, ELISA, and immunoblot assays, with the mean of technical replicates used as a single biological replicate value for final statistical analysis. The exact n value, statistical test, and *post-hoc* method for each experiment are specified in the corresponding figure legends. A two-sided *P* value < 0.05 was considered statistically significant for all tests.

## Results

### ST2^+^Treg subsets are increased in the hearts of DCM mice and exhibit increased expression of Areg

**Figure 1:**
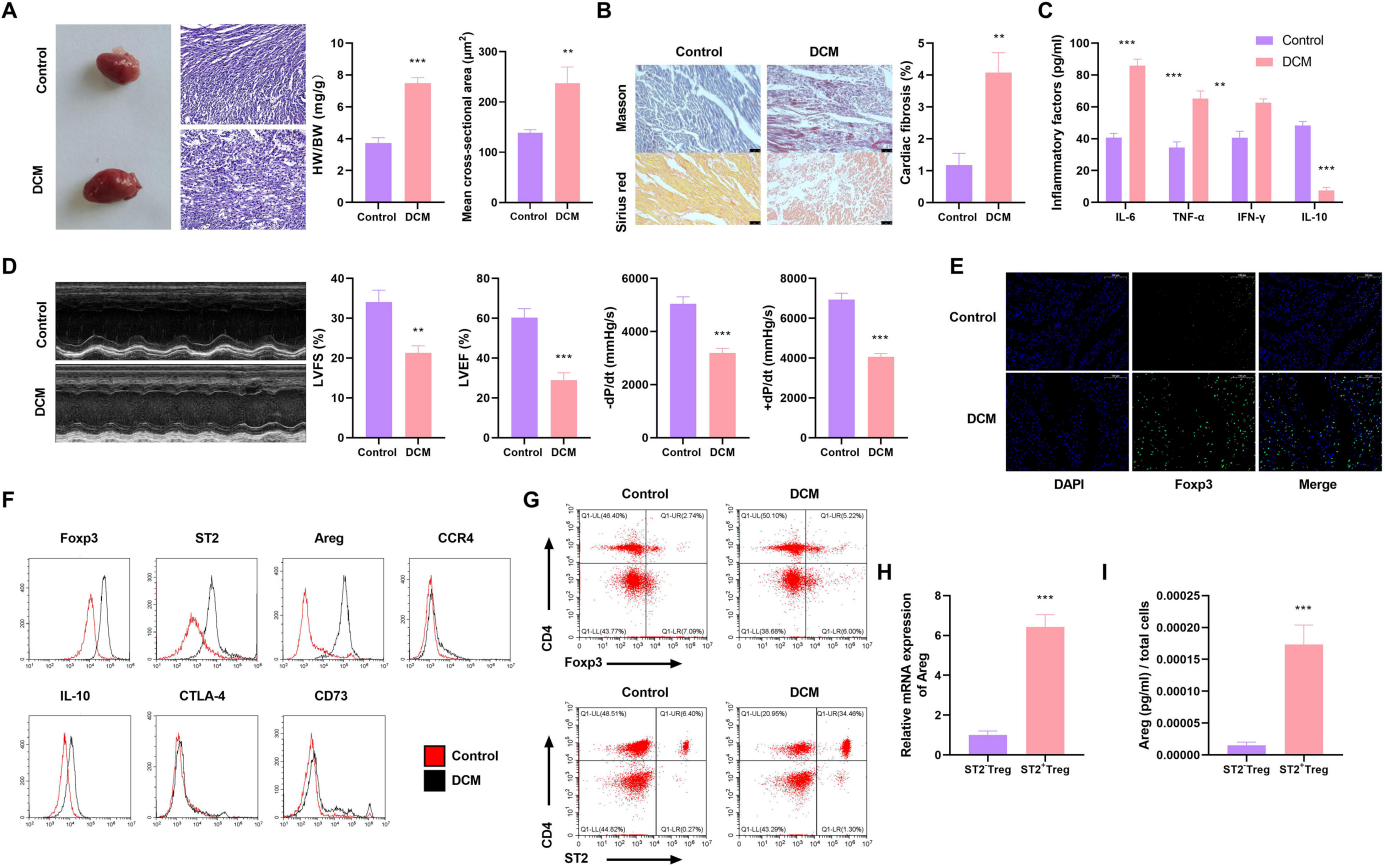
ST2^+^Treg cells are increased in the hearts of DCM mice and exhibit increased expression of Areg. **(A-I)** A DCM mouse model was generated, and their serum samples or heart tissues were harvested for further analyses and the isolation of Treg cells. **(A)** Representative images and H&E staining of mouse hearts, the HW/BW ratio, and the mean cross-sectional area of left ventricular myocardium. **(B)** Representative Masson’s trichrome and Sirius red staining of mouse hearts and quantification of cardiac fibrosis. **(C)** ELISA with serum samples and quantification of IL-6, TNF-α, IFN-γ, and IL-10 secretion levels. **(D)** The echocardiographic detection of DCM mice and sham controls and evaluation of LVFS, LVEF, -dP/dt, and +dP/dt. **(E)** Immunofluorescence staining with the heart tissues using an anti-Foxp3 antibody. **(F, G)** Flow cytometry with the isolated Treg cells from mouse hearts. **(H, I)** Areg mRNA expression by quantitative PCR and its protein levels by ELSIA in ST2^+^Treg and ST2^-^Treg cells. Data are representative of n = 5 mice per group. For histological analysis, N = 3–5 sections per heart were quantified and averaged to obtain a single value per mouse. Statistical test: unpaired two-tailed Student’s t-test. **P < 0.01, ***P < 0.001 vs. Control.

To study the differentiation and role of Treg cells in myocardial fibrosis under DCM, we generated a DCM mouse model. The DCM mice exhibited increased HW/BW ratio and cross-sectional area of left ventricular myocardium compared with control mice (**Figure 1A**). The DCM mice showed enhanced myocardial fibrosis, evaluated by Masson’s trichrome staining and Sirius red staining (**Figure 1B**). The DCM mice also displayed increased serum levels of pro-inflammatory cytokines (IL-6, TNF-α, and IFN-γ) and decreased secretion of anti-inflammatory factor IL-10 (**Figure 1C**), indicating the enhanced inflammation in the DCM mice. Moreover, the echocardiographic detection showed that the DCM mice presented diminished LVFS, LVEF, -dP/dt, and +dP/dt compared with age-matched control mice (**Figure 1D**). These data support the successful generation of the DCM mouse model.

To clarify the involvement of Treg cells in DCM myocardial fibrosis, we performed immunofluorescence with the heart tissues and flow cytometry with isolated Treg cells from mouse hearts. Immunofluorescence staining revealed a significant increase in the number of Treg cells in the hearts of DCM mice, as presented by the elevated expression of Foxp3 (the canonical lineage marker of Treg cells) in DCM cardiac tissues compared with sham controls (**Figure 1E**). The increase in cardiac Foxp3^+^ Treg cell proportion was further confirmed by flow cytometry, with the complete gating strategy provided in Supplementary Material (**Supplementary Figure 1**). Intriguingly, flow cytometry results showed that the proportion of ST2^hi^Areg^hi^ double-positive Treg cells was significantly increased in the cardiac tissues of DCM mice compared with sham controls, with representative flow plots shown in **Figures 1F, G**. The positive gating thresholds for ST2 and Areg were set based on FMO controls, as described in the Materials and Methods section. Notably, sorted ST2^hi^ Treg cells had significantly higher levels of Areg mRNA and protein than ST2-Treg counterparts (**Figures 1H, I**). Thus, the cardiac tissues of the DCM model mice have enhanced proportion of ST2^hi^Areg^hi^ Treg cells.

### DBC1 is upregulated in the hearts of DCM mice and promotes myocardial fibrosis

**Figure 2:**
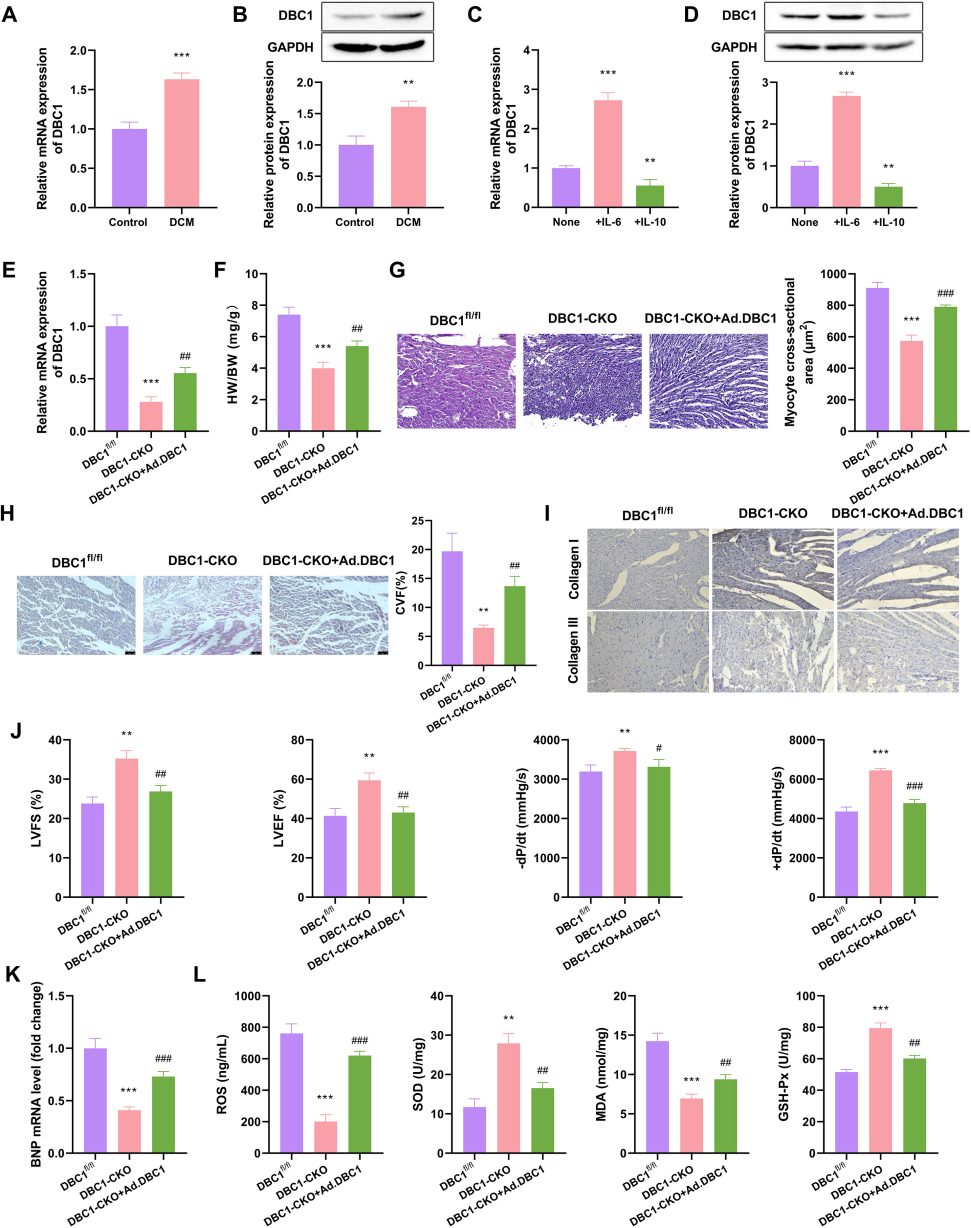
The expression of DBC1 is elevated in the hearts of DCM mice, contributing to the progression of myocardial fibrosis. **(A, B)** Quantitative PCR and immunoblot assays with the heart tissues of Control mice and DCM mice and quantification of DBC1 mRNA and protein levels. Statistical test: unpaired two-tailed Student’s t-test. **(C, D)** Quantification of DBC1 mRNA and protein levels in the heart tissues of DCM mice treated with vehicle, IL-6, or IL-10. **(E-L)** DBC1^fl/fl^ or DBC1-CKO C57BL/6 mice were utilized to generate DCM mouse models accompanied with or without myocardial spot injection of DBC1 adenovirus (Ad.DBC1), followed by the collection of mouse hearts. **(E)** Quantification of DBC1 mRNA in mouse heart tissues. **(F, G)** The HW/BW ratio and the mean cross-sectional area of left ventricular myocardium. **(H)** Representative Masson staining of mouse hearts and quantification of collagen volume fraction (CVF)%. **(I)** Representative immunohistochemical staining of collagen I and collagen III in mouse hearts. **(J)** The echocardiographic detection of DCM mice and evaluation of LVFS, LVEF, -dP/dt, and +dP/dt. **(K)** Quantitative PCR of BNP mRNA in mouse heart tissues. **(L)** The levels of ROS, SOD, MDA, and GSH-Px in mouse heart tissues. Data are representative of n = 5 mice per group. For histological analysis, N = 3–5 sections per heart were quantified and averaged to obtain a single value per mouse. Statistical test: one-way ANOVA with Bonferroni *post-hoc* test. **P < 0.01, ***P < 0.001 vs. Control, None, or DBC1^fl/fl^; ^#^*P* < 0.05, ^##^*P* < 0.01, ^###^*P* < 0.001 vs. DBC1-CKO.

It has been reported that DBC1 can interact with the Treg-specific transcription factor Foxp3, thereby diminishing the immunosuppressive function of Treg cells during inflammatory conditions (17). We thus investigated DBC1 expression in DCM mice and its effect on myocardial fibrosis. Quantitative PCR and immunoblot assays confirmed that the DCM mice exhibited increased expression of DBC1 at both mRNA and protein in their hearts compared with sham controls (**Figures 2A, B**). When we used pro-inflammatory cytokine IL-6 and anti-inflammatory factor IL-10 to treat DCM mice, we confirmed that IL-6 strongly enhanced DBC1 expression in DCM cardiac tissues, while IL-10 significantly downregulated the mRNA and protein levels of DBC1 in the hearts of DCM mice (**Figures 2C, D**), demonstrating the implication of DBC1 in inflammatory response.

To determine whether DBC1 is required for myocardial fibrosis in DCM, DBC1^fl/fl^ C57BL/6 mice were utilized to generate a specific DBC1 knockout model of DCM (DBC1-CKO) accompanied with or without myocardial spot injection of DBC1 adenovirus (Ad.DBC1). Quantitative PCR confirmed the reduced expression of DBC1 in the hearts of DBC1-CKO mice compared with DBC1^fl/fl^ mice (**Figure 2E**) Immunoblot assay confirmed the specific knockout of DBC1 in Treg cells (**Supplementary Figure 2**). Furthermore, Ad.DBC1 injection significantly elevated DBC1 expression in DBC1-CKO DCM mice (**Figure 2E**). By contrast, DBC1 downregulation reduced the HW/BW ratio and mean cross-sectional area of left ventricular myocardium in DCM mice, which could be remarkably reversed by Ad.DBC1 injection (**Figures 2F, G**). Notably, DBC1 downregulation attenuated myocardial fibrosis in DCM mice, as evidenced by the decrease in the ratio of collagen volume fraction (CVF) and the expression of collagen I and collagen III in mouse heart tissues (**Figures 2H, I**). Moreover, Ad.DBC1 administration strongly abolished the impact by DBC1 downregulation on myocardial fibrosis (**Figures 2H, I**). Via M-echocardiographic detection, we observed that downregulation of DBC1 elevated LVFS, LVEF, -dP/dt, and +dP/dt in DCM mice, whereas these alterations were attenuated by Ad.DBC1 injection (**Figure 2J**). Ad.DBC1 injection also markedly rescued DBC1 downregulation-imposed inhibition in the DCM marker BNP in the hearts of DCM mice (**Figure 2K**). Oxidative stress plays a pivotal role in the pathogenesis of DCM, particularly in the context of myocardial fibrosis (26). Interestingly, reduced DBC1 expression led to a striking decrease in ROS and MDA levels and a clear augmentation in SOD and GSH-Px contents in DCM heart tissues, which were dramatically reversed by Ad.DBC1 injection (**Figure 2L**), confirming the promotion of DBC1 in cardiac oxidative stress in DCM mice. Together, these data indicate that upregulated DBC1 in the hearts of DCM mice accelerates myocardial fibrosis.

### ST2^hi^Treg subsets increase myocardial fibrosis in DCM mice by producing Areg

**Figure 3:**
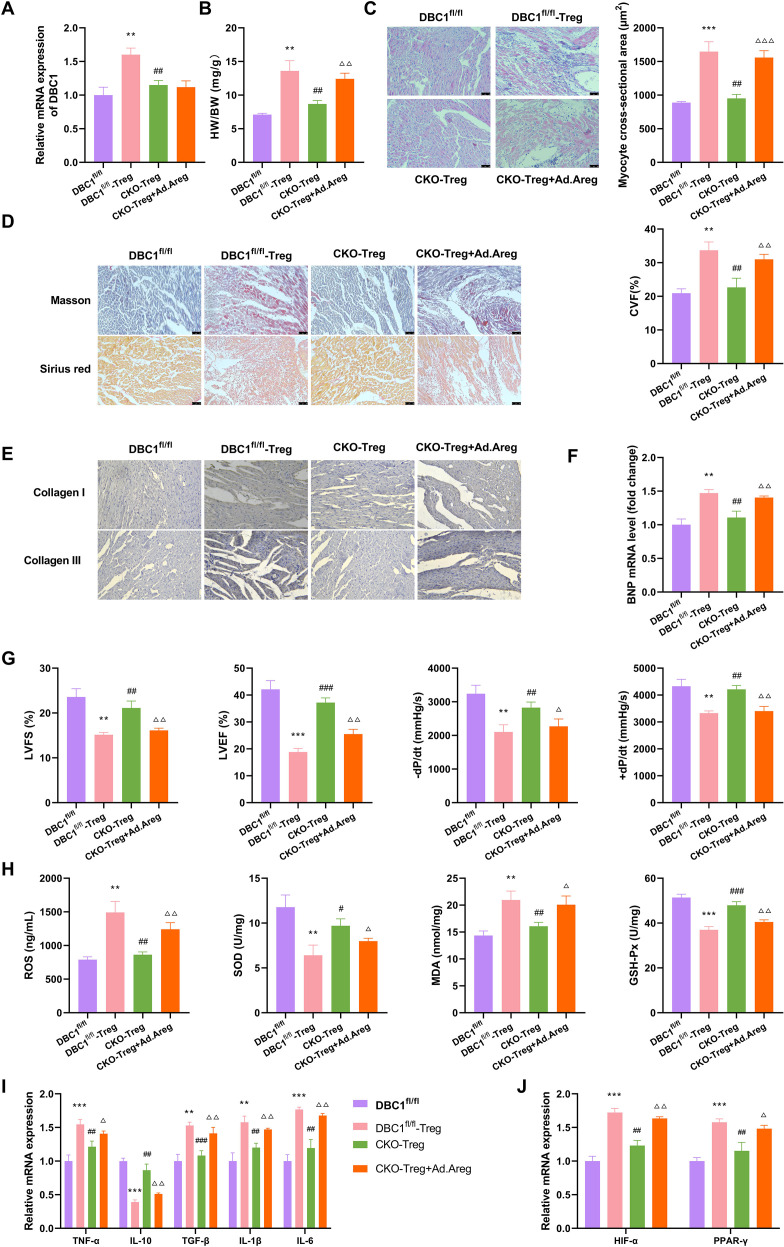
ST2^hi^Areg^hi^ Treg subsets promote myocardial fibrosis in DCM mice by producing Areg. **(A-J)** ST2^hi^ Treg cells were isolated from the cardiac tissues of DBC1^fl/fl^ DCM mice, or DBC1-CKO DCM mice, and adoptively transferred into DCM model mice via tail vein injection (10^6^ cells/mouse), followed by myocardial injection of empty control adenovirus (Ad-NC) or Areg-encoding adenovirus (Ad.Areg). **(A)** Quantification of DBC1 mRNA in mouse heart tissues. **(B, C)** The HW/BW ratio, H&E staining, and the mean cross-sectional area of left ventricular myocardium. **(D)** Representative Masson’s trichrome and Sirius red staining of mouse hearts and quantification of collagen volume fraction (CVF)%. **(E)** Representative immunohistochemical staining of collagen I and collagen III in mouse hearts. **(F)** Quantitative PCR of BNP mRNA in mouse heart tissues. **(G)** The echocardiographic detection of DCM mice and evaluation of LVFS, LVEF, -dP/dt, and +dP/dt. **(H)** The levels of ROS, SOD, MDA, and GSH-Px in mouse heart tissues. **(I)** The mRNA levels of TNF-α, IL-10, TGF-β, IL-1β, and IL-6 in mouse heart tissues. **(J)** The mRNA expression of HIF-1α and PPAR-γ in the hearts of DCM mice. Data are representative of n = 5 mice per group. For histological analysis, N = 3–5 sections per heart were quantified and averaged to obtain a single value per mouse. Statistical test: two-way ANOVA with Bonferroni *post-hoc* test. **P < 0.01, ***P < 0.001 vs. DBC1^fl/fl^; ^#^*P* < 0.05, ^##^*P* < 0.01, ^###^*P* < 0.001 vs. DBC1^fl/fl^-Treg; ^Δ^*P* < 0.05, ^ΔΔ^*P* < 0.01, ^ΔΔΔ^*P* < 0.001 vs. CKO-Treg.

Our aforementioned data indicate that the ST2^hi^Areg^hi^ Treg subsets are increased in the hearts of DCM mice. To elucidate the biological impact of these cells on myocardial fibrosis under DCM, we performed adoptive transfer of ST2^+^Treg cells into DCM mice. ST2^hi^Treg cells were isolated from the cardiac tissues of DBC1^fl/fl^ (DBC1^fl/fl^-Treg) or DBC1-CKO (CKO-Treg) DCM mice and adoptively transferred into DCM model mice via tail vein injection, followed by myocardial spot injection with or without Areg adenovirus (Ad.Areg). Adoptive transfer of DBC1^fl/fl^-Treg significantly increased DBC1 expression in the hearts of DBC1^fl/fl^ DCM mice, while CKO-Treg caused a weaker increase in DBC1 expression compared with the DBC1^fl/fl^-Treg group (**Figure 3A**). Strikingly, adoptive transfer of DBC1^fl/fl^-Treg increased the HW/BW ratio (**Figure 3B**), mean cross-sectional area of left ventricular myocardium (**Figure 3C**), the cardiac CVF ratio (**Figure 3D**), cardiac collagen I and collagen III expression (**Figure 3E**), and cardiac BNP mRNA expression (**Figure 3F**), as well as weakened cardiac LVFS, LVEF, -dP/dt, and +dP/dt (**Figure 3G**) in DCM mice. Furthermore, DBC1^fl/fl^-Treg strongly enhanced cardiac oxidative stress, as indicated by the increase in ROS and MDA levels and the decrease in SOD and GSH-Px contents in DCM mice with adoptive transfer of DBC1^fl/fl^-Treg (**Figure 3H**). DBC1^fl/fl^-Treg also elevated the mRNA levels of TNF-α, TGF-β, IL-1β, and IL-6 but reduced IL-10 mRNA expression in the hearts of DCM mice (**Figure 3I**), suggesting the promotion of DBC1^fl/fl^-Treg in inflammatory response. Previous evidence has highlighted the critical modulation of the HIF-1α-PPAR-γ axis in Treg cell differentiation (18). Intriguingly, our data showed that adoptive transfer of DBC1^fl/fl^-Treg upregulated the mRNA expression of HIF-1α and PPAR-γ in the hearts of DCM mice (**Figure 3J**), suggesting the facilitation of DBC1^fl/fl^-Treg in the activation of the HIF-1α-PPAR-γ axis. These data together demonstrate that adoptive transfer of ST2^hi^Treg subsets from WT DCM mice enhances myocardial fibrosis and oxidative stress of DCM model mice.

Relative to the DBC1^fl/fl^-Treg group, adoptive transfer of CKO-Treg had less pronounced effects on the HW/BW ratio and mean cross-sectional area of left ventricular myocardium (**Figures 3B, C**), cardiac fibrosis (**Figures 3D, E**), cardiac BNP mRNA expression (**Figure 3F**), cardiac echocardiographic alterations (**Figure 3G**), cardiac oxidative stress (**Figure 3H**), cardiac inflammatory response (**Figure 3I**), as well as the activation of the HIF-1α-PPAR-γ axis (**Figure 3J**) in DCM mice, indicating that ST2^+^Treg subsets from DBC1^fl/fl^ mice have stronger impact on promoting cardiac fibrosis than ST2^+^Treg cells from DBC1-CKO DCM. More interestingly, although myocardial spot injection of Areg did not significantly change the expression levels of DBC1 in DCM mouse hearts (**Figure 3A**), it really aggravated these effects of CKO-Treg on cardiac phenotypic alterations in DCM mice (**Figures 3B-J**). Collectively, these results suggest that ST2^hi^Areg^hi^ Treg subsets exhibit increased DBC1 expression and promote myocardial fibrosis by producing Areg. Mechanistically, the Ad.Areg rescue experiment confirms that Areg is the key downstream effector molecule mediating the pro-fibrotic effect of ST2^hi^ Tregs: DBC1 deficiency in Tregs reduced Areg production and thus attenuated the pro-fibrotic phenotype, while myocardial overexpression of Areg via Ad.Areg fully reversed this protective effect, directly demonstrating that Areg is sufficient to drive the pro-fibrotic effect of ST2^hi^ Tregs *in vivo*.

### DBC1 mediates the differentiation of Treg cells into the ST2^hi^Areg^hi^ subgroup in DCM

**Figure 4:**
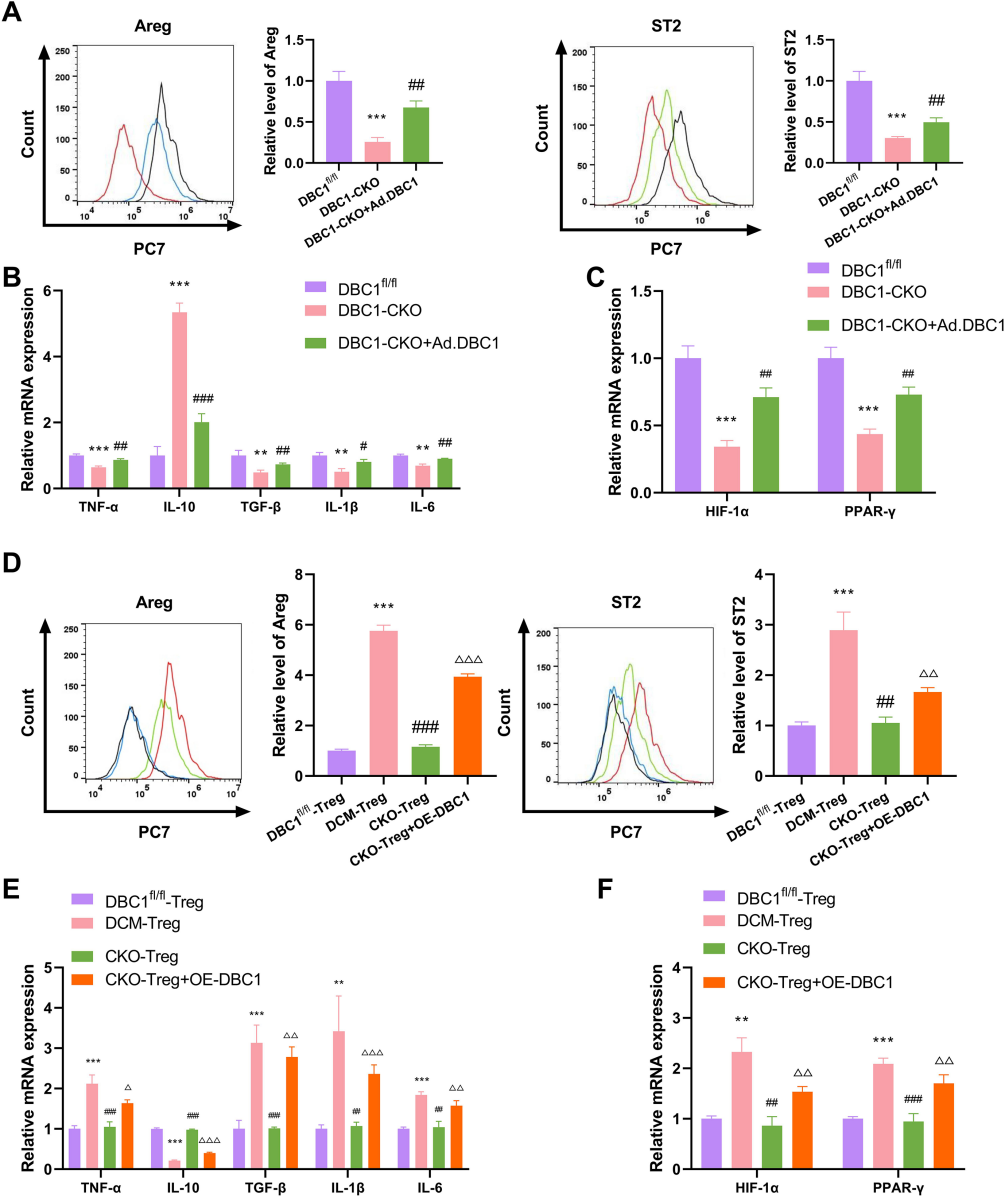
DBC1 induces Treg cell differentiation to ST2^hi^Areg^hi^ subsets in the hearts of DCM mice. **(A-C)** DBC1^fl/fl^ or DBC-CKO C57BL/6 mice were utilized to generate DCM mouse models accompanied with or without myocardial spot injection of DBC1 adenovirus (Ad.DBC1), followed by the collection of mouse hearts. **(A)** Flow cytometry for Areg and ST2 expression in cardiac single-cell suspension. **(B)** The mRNA levels of TNF-α, IL-10, TGF-β, IL-1β, and IL-6 in mouse heart tissues. **(C)** The mRNA expression of HIF-1α and PPAR-γ in mouse heart tissues. **(D-F)** Treg cells were isolated from the hearts of DBC1^fl/fl^ mice (DBC1^fl/fl^-Treg), DBC1^fl/fl^ DCM mice (DCM-Treg), and DBC1-CKO DCM mice (CKO-Treg). The CKO-Treg cells were subjected to transfection with OE-DBC1. **(D)** Flow cytometry for Areg and ST2 expression in isolated Treg cells. **(E)** The mRNA levels of TNF-α, IL-10, TGF-β, IL-1β, and IL-6 in isolated Treg cells. **(F)** The mRNA expression of HIF-1α and PPAR-γ in isolated Treg cells. Statistical test: one-way ANOVA with Bonferroni *post-hoc* test. **P < 0.01, ***P < 0.001 vs. DBC1^fl/fl^ or DBC1^fl/fl^-Treg; ^#^*P* < 0.05, ^##^*P* < 0.01, ^###^*P* < 0.001 vs. DBC1-CKO or DCM-Treg; ^Δ^*P* < 0.05, ^ΔΔ^*P* < 0.01, ^ΔΔΔ^*P* < 0.001 vs. CKO-Treg.

Given that DBC1 contributes to myocardial fibrosis and is upregulated in ST2^hi^Treg cells from DCM mice, we sought to study the role of DBC1 in Treg cell differentiation. To this end, we performed flow cytometry assay with the cardiac single-cell suspension of DBC1^fl/fl^ mice, DBC1-CKO DCM mice, or DBC1-CKO DCM mice with Ad.DBC1 injection. These results revealed that the proportion of ST2^hi^Areg^hi^ Treg cells in the cardiac tissues of DBC1-CKO DCM mice was significantly lower than that in DBC1^fl/fl^ DCM mice, while myocardial injection of Ad.DBC1 significantly reversed this reduction, with representative flow plots shown in **Figure 4A**. The DBC1-CKO DCM mice displayed reduced mRNA levels of TNF-α, TGF-β, IL-1β, and IL-6, and increased expression of IL-10 mRNA expression, as well as decreased mRNA levels of HIF-1α and PPAR-γ in their hearts, which could be strongly reversed by Ad.DBC1 injection (**Figures 4B, C**). We also isolated Treg cells from the hearts of DBC1^fl/fl^ mice (DBC1^fl/fl^-Treg), DCM mice (DCM-Treg) and DBC1-CKO DCM mice (CKO-Treg). The DCM-Treg cells presented higher levels of Areg and ST2, and the CKO-Treg cells had decreased Areg and ST2 expression compared with the DCM-Treg cells (**Figure 4D**). Transfection of CKO-Treg cells with a DBC1 expression construct (OE-DBC1) significantly elevated the expression of Areg and ST2 (**Figure 4D**). Compared with the DBC1^fl/fl^-Treg group, the DCM-Treg cells had higher mRNA levels of TNF-α, TGF-β, IL-1β, IL-6, HIF-1α, and PPAR-γ and lower mRNA expression of IL-10 (**Figures 4E, F**). The CKO-Treg cells exhibited decreased mRNA expression of TNF-α, TGF-β, IL-1β, IL-6, HIF-1α, and PPAR-γ and elevated IL-10 expression compared with the DCM-Treg cells, whereas OE-DBC1 reversed these alterations (**Figures 4E, F**). These data suggest that DBC1 has the ability to induce Treg cell differentiation to ST2^hi^Areg^hi^ subsets in the hearts of DCM mice.

### ST2^hi^Treg subsets promote the *in vitro* viability, migration, and fibrosis of mouse CFs through the paracrine secretion of Areg

**Figure 5:**
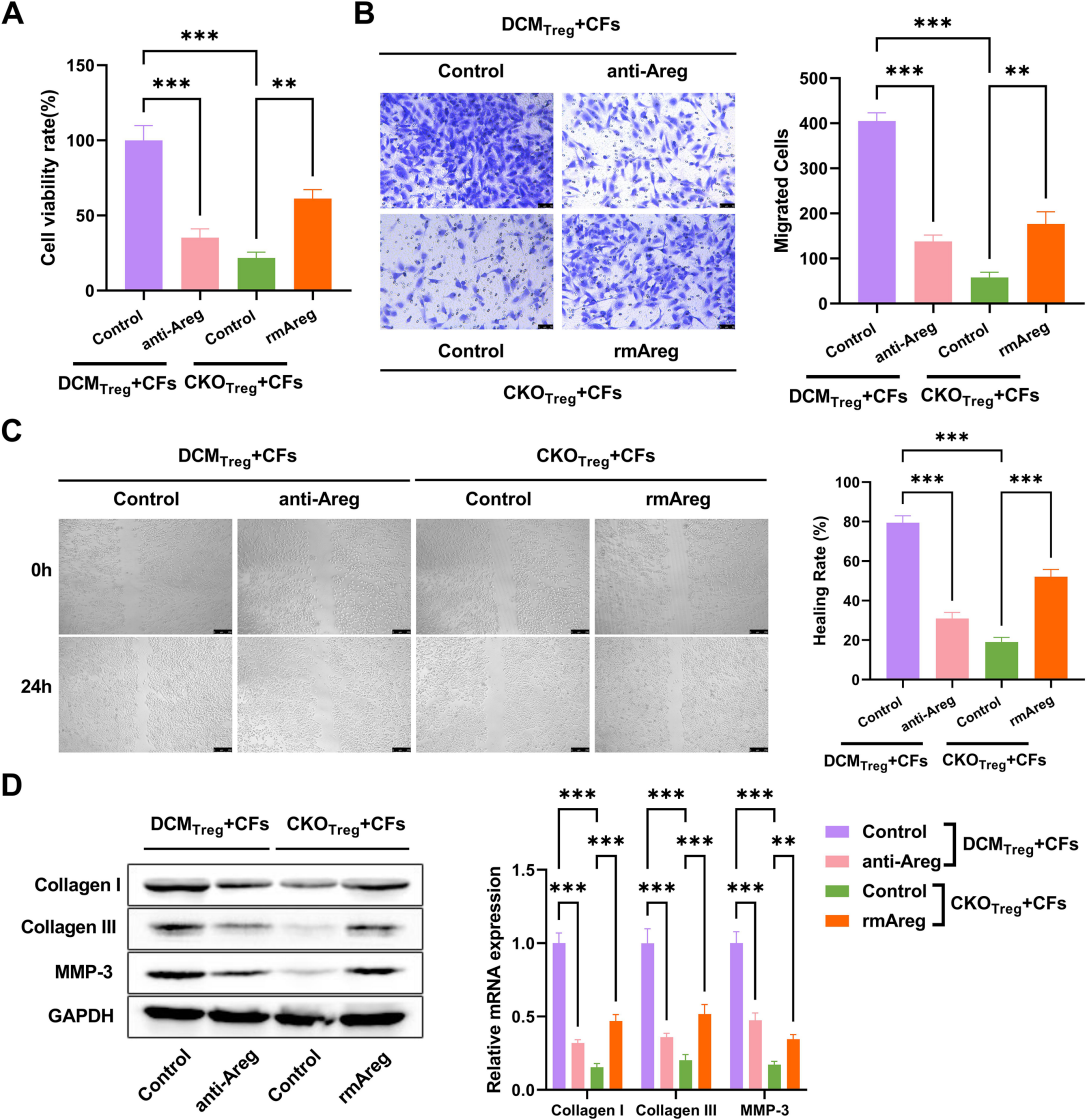
ST2^hi^Treg cells accelerate the viability, migration, and fibrosis of mouse CFs by secreting Areg. **(A-D)** Mouse CFs were co-cultured with ST2^hi^Treg cells isolated from the cardiac tissues of DCM (DCM_Treg_) under treatment with or without an anti-Areg antibody or ST2^hi^Treg cells from DBC1-CKO (CKO_Treg_) DCM mice under administration with or without recombinant mouse Areg (rmAreg). **(A)** Viability of co-cultured CFs by CCK-8 assay. **(B, C)** Migration of co-cultured CFs by transwell and wound healing assays. **(D)** Expression of collagen I, collagen III, and MMP-3 in co-cultured CFs. Statistical test: two-way ANOVA with Bonferroni *post-hoc* test. **P < 0.01, ***P < 0.001 vs. DCM_Treg_+CFs+Control.

The *in vivo* studies suggest that ST2^hi^Areg^hi^ Treg subsets enhance cardiac fibrosis in DCM mice by producing Areg. Accordingly, we determined whether ST2^hi^Treg subsets regulate the function of cardiac fibroblasts (CFs) *in vitro*. To address this, mouse CFs were co-cultured with ST2^hi^Treg cells isolated from the cardiac tissues of DBC1^fl/fl^ (DCM_Treg_) or DBC1-CKO (CKO_Treg_) DCM mice under treatment with or without an anti-Areg antibody or recombinant mouse Areg (rmAreg). Compared with the DCM_Treg_ group, co-culture of CFs with CKO_Treg_ resulted in suppressed viability (**Figure 5A**) and migratory ability (**Figures 5B, C**) in CFs as well as decreased protein levels of collagen I, collagen III, and MMP-3 (**Figure 5D**). In the DCM_Treg_+CFs co-culture system, treatment of the anti-Areg antibody significantly decreased cell viability (**Figure 5A**), migratory ability (**Figures 5B, C**), and the expression of collagen I, collagen III, and MMP-3 (**Figure 5D**) in CFs. On the other hand, in the CKO_Treg_+CFs co-culture system, replenishment of Areg by rmAreg remarkably enhanced cell viability (**Figure 5A**), migration (**Figures 5B, C**), and the expression of collagen I, collagen III, and MMP-3 (**Figure 5D**) in CFs. Thus, ST2^hi^Areg^hi^ Treg cells accelerate the viability, migration, and fibrosis of mouse CFs by secreting Areg.

### DBC1 drives the differentiation of Treg cell subsets through the HIF-1α-PPAR-γ axis

**Figure 6:**
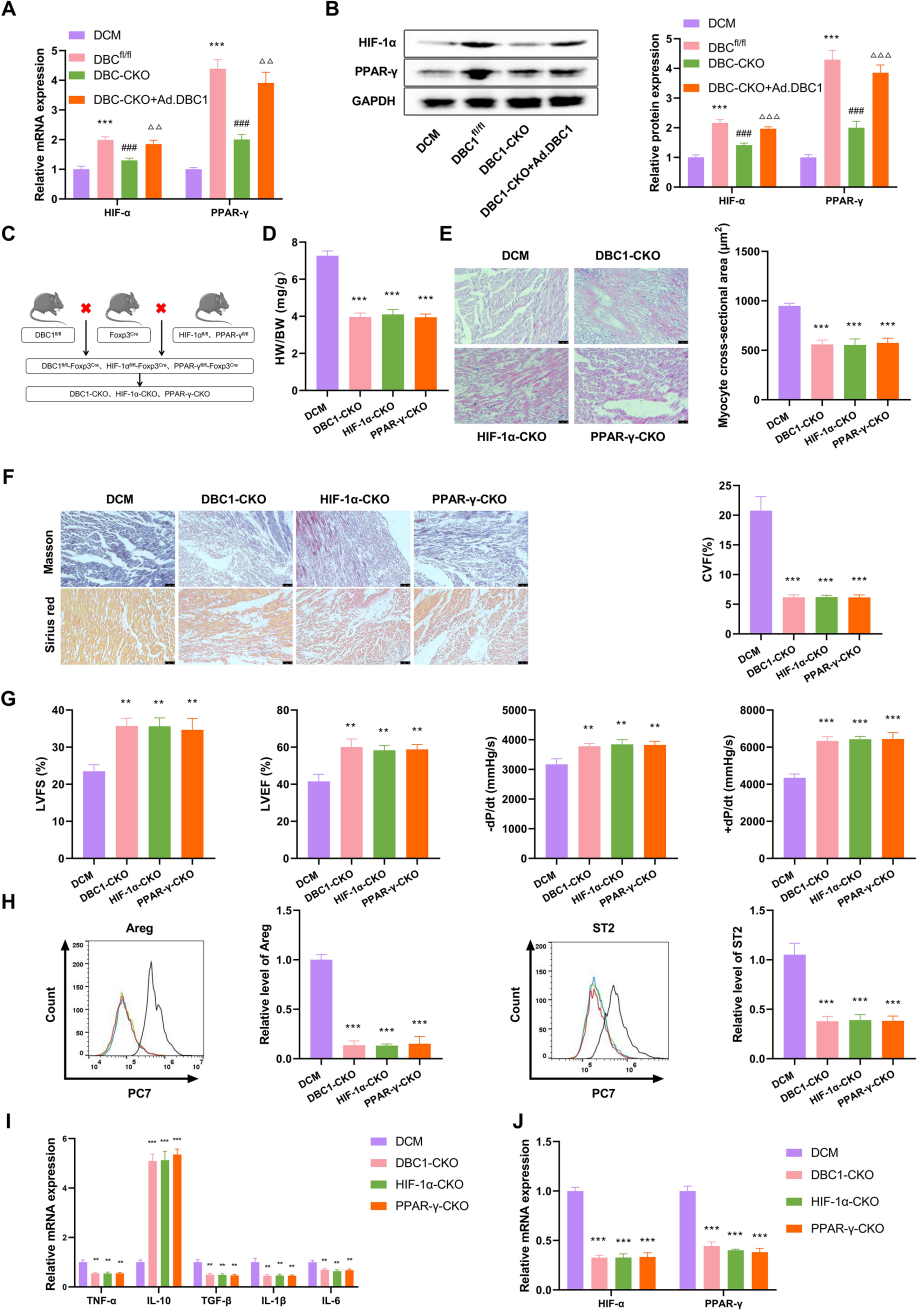
The DBC1-HIF-1α-PPAR-γ axis affects Treg cell differentiation to ST2^hi^Areg^hi^ subsets and myocardial fibrosis in DCM mice. **(A, B)** Quantification of the mRNA and protein levels of HIF-1α and PPAR-γ in mouse hearts. **(C)** Schematic of the generation of DBC1-CKO, HIF-1α-CKO, and PPAR-γ-CKO mice. **(D-J)** DBC1-CKO, HIF-1α-CKO, and PPAR-γ-CKO mice were established and their hearts were harvested, followed by the determination of the HW/BW ratio **(D)**, the mean cross-sectional area of left ventricular myocardium **(E)**, the collagen volume fraction (CVF)% **(F)**, LVFS, LVEF, -dP/dt, and +dP/dt **(G)**, Areg and ST2 expression **(H)**, the mRNA levels of TNF-α, IL-10, TGF-β, IL-1β, IL-6, HIF-1α, and PPAR-γ **(I, J)**. Data are representative of n = 5 mice per group. For histological analysis, N = 3–5 sections per heart were quantified and averaged to obtain a single value per mouse. Statistical test: one-way ANOVA with Bonferroni *post-hoc* test. **P < 0.01, ***P < 0.001 vs. DCM; ^###^*P* < 0.001 vs. DBC1^fl/fl^; ^ΔΔ^*P* < 0.01, ^ΔΔΔ^*P* < 0.001 vs. DBC1-CKO.

Considering the critical influence of the HIF-1α-PPAR-γ axis in Treg cell differentiation (18), and since our data have suggested the regulation of DBC1 in the axis (**Figure 4F**), we finally investigated whether the HIF-1α-PPAR-γ axis is responsible for DBC1-mediated Treg cell differentiation to ST2^hi^Areg^hi^ subsets. The DBC1^fl/fl^ DCM mice presented increased expression of HIF-1α and PPAR-γ at both mRNA and protein in their hearts compared with the DBC1^fl/fl^-Normal mice (**Figures 6A, B**). The DBC1-CKO DCM mice exhibited decreased HIF-1α and PPAR-γ expression compared to the DCM mice, while Ad.DBC1 injection significantly elevated their expression (**Figures 6A, B**), reinforcing the notion that DBC1 can activate the HIF-1α-PPAR-γ axis in the hearts of DCM mice.

Further, we employed the DBC1-CKO, HIF-1α-CKO, and PPAR-γ-CKO DCM mice, which were generated by a mating method illustrated in **Figure 6C** and deleted DBC1, HIF-1α, or PPAR-γ in their Treg cells, respectively, to establish a DCM mouse model. Surprisingly, the HW/BW ratio (**Figure 6D**), mean cross-sectional area of left ventricular myocardium (**Figure 6E**), the cardiac CVF ratio (**Figure 6F**), and cardiac LVFS, LVEF, -dP/dt, and +dP/dt (**Figure 6G**) in DBC1-CKO, HIF-1α-CKO, and PPAR-γ-CKO DCM mice were comparable. Furthermore, DBC1-CKO, HIF-1α-CKO, and PPAR-γ-CKO DCM mice showed a comparable and significant reduction in the proportion of cardiac ST2^hi^Areg^hi^ Treg cells, with representative flow plots shown in **Figure 6H**. Additionally, the knockout of DBC1, HIF-1α, or PPAR-γ in Treg cells led to comparable alterations in the mRNA expression of TNF-α, IL-10, TGF-β, IL-1β, IL-6, HIF-1α, and PPAR-γ in mouse hearts (**Figures 6I, J**). These observations underscore the critical role of the DBC1-HIF-1α-PPAR-γ axis in Treg cell differentiation to ST2^hi^Areg^hi^ subsets and myocardial fibrosis in DCM mice.

**Figure 7:**
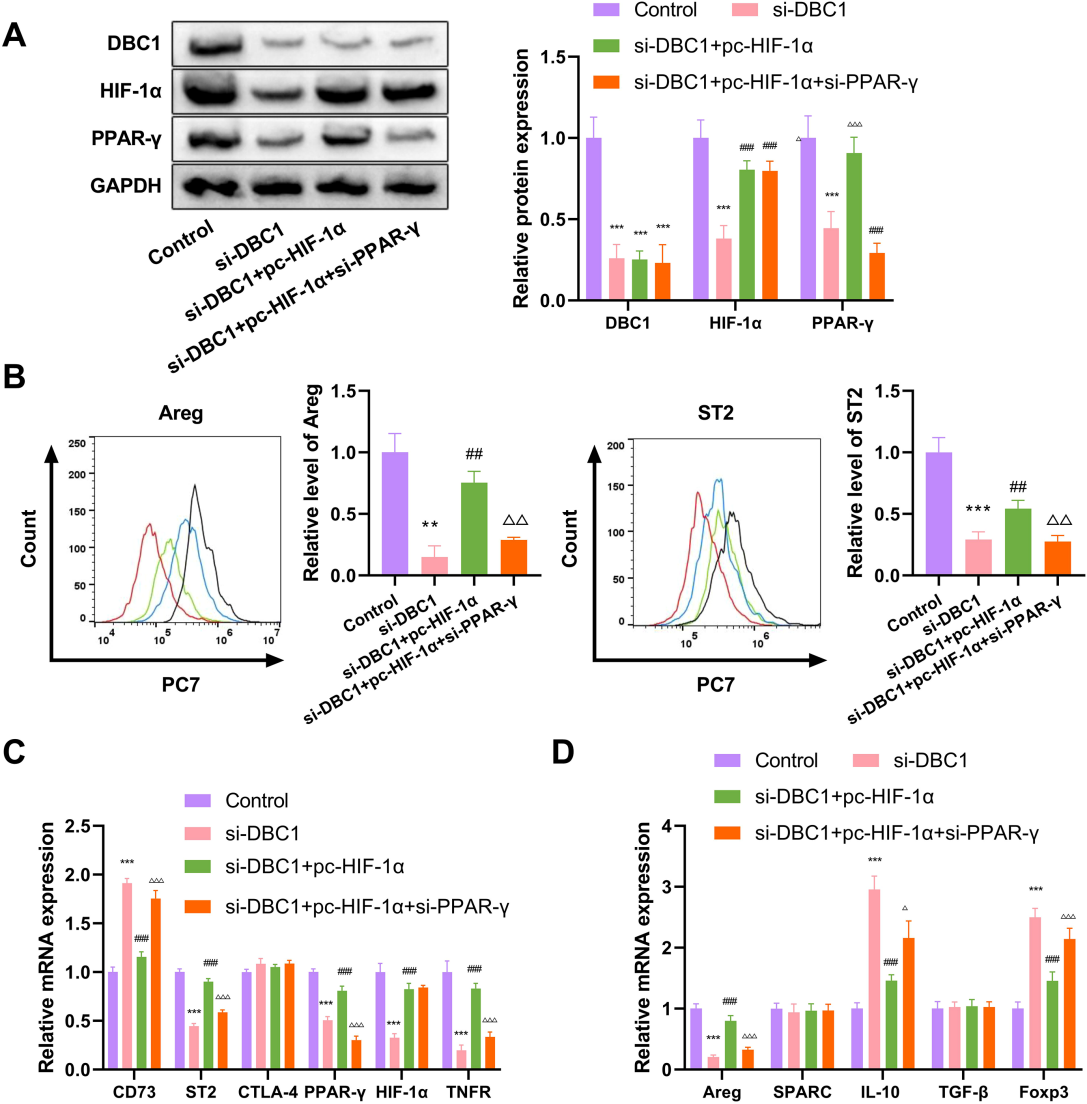
DBC1 regulates Treg cell differentiation to ST2^hi^Areg^hi^ subsets through the HIF-1α-PPAR-γ axis *in vitro*. **(A)** Western blot analysis of protein expressions of DBC1, HIF-1α and PPAR-γ. **(B-D)** ST2^hi^Treg cells were isolated from the hearts of WT DCM mice and subjected to transfection with si-DBC1, si-DBC1+pc-HIF-1α, or si-DBC1+pc-HIF-1α+si-PPAR-γ constructs, followed by the assessment of Areg and ST2 expression **(B)** and the mRNA levels of related factors (C and D). Statistical test: one-way ANOVA with Bonferroni *post-hoc* test. **P < 0.01, ***P < 0.001 vs. Control; ^##^*P* < 0.01, ^###^*P* < 0.001 vs. si-DBC1; ^Δ^*P* < 0.05, ^ΔΔ^*P* < 0.01, ^ΔΔΔ^*P* < 0.001 vs. si-DBC1+pc-HIF-1α.

To support these findings *in vivo*, ST2^hi^Treg cells were isolated from the hearts of DBC1^fl/fl^ DCM mice and subjected to transfection with si-DBC1, si-DBC1+pc-HIF-1α, or si-DBC1+pc-HIF-1α+si-PPAR-γ constructs (**Figure 7A**). DBC1 depletion in ST2^hi^Treg cells led to a significant downregulation in Areg and ST2 expression (**Figure 7B**). Co-transfection of pc-HIF-1α increased Areg and ST2 expression in DBC1-depleted ST2^hi^Treg cells, while si-PPAR-γ significantly abolished the impact (**Figure 7B**). Consistently, quantitative PCR assays showed that the depletion of DBC1 decreased the mRNA levels of ST2, PPAR-γ, HIF-1α, TNFR, and Areg and elevated the mRNA expression of CD73, IL-10, and Foxp3 in ST2^hi^Treg cells (**Figures 7C, D**). Co-transfection of pc-HIF-1α significantly reversed these mRNA expression changes induced by DBC1 depletion, which could also be abolished by si-PPAR-γ (**Figures 7C, D**). However, the mRNA levels of CTLA-4, SPARC, and TGF-β in ST2^hi^Treg cells did not alter following the different transfections (**Figures 7C, D**). Collectively, these findings demonstrate the regulation of the DBC1-HIF-1α-PPAR-γ axis in Treg cell differentiation to ST2^hi^Areg^hi^ subsets.

## Discussion

Treg cells can differentiate into various subpopulations with distinct functions, including immunosuppressive and tissue repair properties. Different subtypes of Treg cells play distinct functions by secreting various factors, such as anti-inflammatory cytokines (e.g. IL-10, IL-35) and tissue repair molecules (e.g. Areg, CD73) (8). In pathological inflammatory conditions, Treg cells frequently display instability and undergo changes in their functional differentiation into different subpopulations (10). Among these subpopulations, ST2^hi^ and Areg^hi^ subgroups have been highlighted to contribute to the regenerative and pro-fibrotic properties of Treg cells (11, 27, 28). The roles of these different Treg cell subsets in human diseases have been increasingly recognized, demonstrating their involvement in conditions such as autoimmune disorders, cancer, and metabolic syndromes (10, 29). Due to their capacity to regulate myocardial fibrosis, inflammation, and oxidative stress, Treg cells have become a promising focus of research for the treatment of DCM (9, 30). In our current study, we show an increase in ST2^hi^Treg cells in the hearts of DCM mice, which is accompanied by elevated expression of Areg. By utilizing adoptive cell transfer therapy, an innovative therapeutic approach that has shown considerable promise in disease treatment (31–33), we demonstrate that ST2^hi^Areg^hi^ Treg subsets promote myocardial fibrosis in DCM mice by producing Areg. Activated fibroblasts are key cellular effectors in cardiac fibrosis, acting as the primary source of extracellular matrix proteins (4). Using *in vitro* functional analyses, we unveil that ST2^hi^Areg^hi^ Treg cells have the ability to enhance the viability, migration, and fibrotic activity of CFs via the paracrine secretion of Areg. These findings suggest that ST2^hi^Areg^hi^ Treg subsets drive maladaptive pathological myocardial fibrosis in the chronic inflammatory microenvironment of DCM, rather than mediating physiological reparative scarring as observed in acute tissue injury settings. Specifically, in our DCM model, ST2^hi^Areg^hi^ Treg-derived Areg directly promoted the proliferation, migration, and pro-fibrotic activation of cardiac fibroblasts, leading to excessive accumulation of collagen I and collagen III, increased myocardial stiffness, and deteriorated left ventricular systolic and diastolic function (as evidenced by significantly reduced LVEF, LVFS, and ± dP/dt in mice receiving adoptive transfer of ST2^hi^Areg^hi^ Tregs). This progressive, maladaptive remodeling is fundamentally distinct from transient reparative scarring, which acts to preserve cardiac wall integrity and prevent cardiac rupture after acute myocardial infarction. These results reconcile the apparent functional duality of ST2^hi^Areg^hi^ Tregs in cardiovascular diseases: while this subset exerts beneficial tissue-reparative effects in the context of acute ischemic injury, it drives pathological fibrosis in the setting of chronic metabolic inflammation such as DCM, deepening our understanding of Treg cell functional heterogeneity in the diabetic heart. It is important to note that Treg subsets exhibit functional plasticity depending on the tissue microenvironment. For instance, CD45RA^+^ and Helios^+^ Tregs, often associated with thymic origin and stable immunosuppressive function, have been implicated in neonatal heart regeneration and cardiomyocyte proliferation via paracrine mechanisms (34). However, in the chronic inflammatory milieu of DCM, Tregs undergo phenotypic shifts, favoring the expansion of effector-like ST2hiAreghi subsets that promote fibrosis rather than regeneration. This aligns with recent findings showing that inflammatory signals such as IL-6 and hyperglycemia drive Treg instability and functional reprogramming toward pro-fibrotic phenotypes (27). Thus, the functional outcome of Treg activity is highly context-dependent, and the absence of CD45RA^+^/Helios^+^ Treg dominance in our model reflects the pathological nature of DCM, contrasting with regenerative models. To further support this pro-fibrotic rather than pro-reparative phenotype conclusion, we assessed a panel of established markers for tissue repair and pathological fibrosis using our existing experimental data: for pro-fibrotic markers, we detected significant upregulation of collagen I, collagen III, and MMP-3 in cardiac fibroblasts co-cultured with ST2^hi^Areg^hi^ Tregs, as well as increased myocardial collagen deposition, HW/BW ratio, and heart failure marker BNP *in vivo*; for tissue repair-related markers, we found no significant changes in the expression of reparative/immunosuppressive molecules (TGF-β, CD73, CTLA-4) in ST2^hi^Areg^hi^ Tregs, while the anti-inflammatory and reparative cytokine IL-10 was significantly downregulated in the hearts of mice receiving ST2^hi^Areg^hi^ Treg adoptive transfer. These data collectively confirm that ST2^hi^Areg^hi^ Tregs in DCM exhibit a pro-fibrotic phenotype, rather than a tissue-reparative phenotype. Understanding the dynamics of Treg cell behavior in DCM could uncover new therapeutic targets for modulating myocardial fibrosis and improving cardiac outcomes in diabetic patients.

DBC1 plays a significant role in the inflammatory response, acting as a key mediator that enhances pro-inflammatory signaling pathways. It has emerged as a potential therapeutic target due to its ability to regulate metabolic syndrome and inflammation (14). Knockdown of DBC1 can attenuate the expression of inflammatory factors in adipocytes, indicating its association with adipose tissue senescence (35). Research has revealed that DBC1 is implicated in several human diseases, including cancer, diabetes, and metabolic disorders (16, 36, 37). Notably, DBC1 has also been found to regulate Treg cell functions, influencing their Foxp3 stability and cell differentiation under inflammatory conditions (17). However, no studies addressed the role of DBC1 in the pathogenesis of DCM. In the present study, we have found that DBC1 is upregulated in the hearts of DCM mice. By generating a specific knockout model of DCM, we show that DBC1 knockout significantly alleviates myocardial fibrosis, improves cardiac function, and reduces oxidative stress and inflammation levels in DCM mice. Importantly, we demonstrate, for the first time, that DBC1 promotes the differentiation of Treg cells into ST2^hi^Areg^hi^ subsets in the cardiac environment of DCM mice. Thus, DBC1 may serve as a pivotal molecule involved in regulating the differentiation and function of Treg cells in the myocardium of DCM. Further investigation into the regulatory mechanisms of DBC1 could provide valuable insights for treatment strategies.

The HIF-1α-PPAR-γ axis is a critical signaling pathway that plays a pivotal role in cellular responses to hypoxia and inflammation. The interaction between HIF-1α and PPAR-γ integrates metabolic and inflammatory responses (38). This axis is involved in the pathogenesis of cardiac hypertrophy in high-fat diet rats (39). Furthermore, the HIF-1α-PPAR-γ axis has been shown to regulate Treg cell function, as HIF-1α and PPAR-γ can impact the ST2^hi^ subgroup differentiation and stability of Treg cells in inflammatory microenvironments (18). In our current study, we have discovered that DBC1 can activate the HIF-1α-PPAR-γ axis in the hearts of DCM mice. More importantly, we demonstrate that DBC1 promotes the differentiation of Treg cells into the ST2^hi^Areg^hi^ subsets through the HIF-1α-PPAR-γ axis *in vivo* and *in vitro*. The discovery of the DBC1-HIF-1α-PPAR-γ axis in Treg cell differentiation provides novel insights into the mechanisms underlying cardiac inflammation and fibrosis in DCM. Targeting this novel cascade may offer new therapeutic strategies for DCM treatment by regulating the immune response and fibrosis in the heart.

In this study, we provide compelling evidence regarding the role of ST2^hi^Areg^hi^ Treg cells and DBC1 in the pathophysiology of DCM. However, several limitations must be acknowledged in this study. The primary constraint is the reliance on murine models, which may not fully recapitulate the complexity of human DCM. Nevertheless, several features of our model align closely with human DCM. Notably, the cardiac dysfunction and fibrosis observed in our model are present at rest, suggesting a more advanced phenotype compared to models where pathology is only unmasked under exercise or pharmacological stress. This may reflect the prolonged metabolic insult induced by high-fat diet and low-dose STZ, leading to sustained inflammation and oxidative stress that drive maladaptive remodeling even in the absence of additional stimuli. Clinical studies have demonstrated that myocardial fibrosis in diabetic patients is characterized by interstitial and perivascular collagen deposition, particularly of collagen I and III, mirroring the fibrotic patterns observed in our model (40). Furthermore, immune cell infiltration, including increased Treg populations in cardiac tissues, has been reported in human diabetic hearts and correlates with fibrosis severity (41). Notably, Areg (amphiregulin) has been detected in elevated levels in the serum of patients with type 2 diabetes and heart failure, and its expression is associated with adverse cardiac remodeling (42). Importantly, the HIF-1α-PPAR-γ axis is evolutionarily conserved and functionally active in human Treg cells, with recent single-cell RNA sequencing studies revealing distinct pro-fibrotic Treg subsets in human failing hearts that express high levels of ST2 (IL1RL1) and AREG (43). These findings suggest that the DBC1-HIF-1α-PPAR-γ-ST2^hi^Areg^hi^ axis may be operational in human DCM, although direct validation is required. The translational relevance of our findings to human pathology requires further validation in clinical samples. Second, while our *in vitro* data using anti-Areg neutralizing antibody confirmed that Areg is necessary for ST2^hi^ Treg-induced pro-fibrotic activation of cardiac fibroblasts, and our *in vivo* Ad.Areg rescue experiment demonstrated that Areg is sufficient to drive the pro-fibrotic effect of ST2^hi^ Tregs, we acknowledge the lack of *in vivo* loss-of-function experiments to directly validate the necessity of Areg for ST2^hi^ Treg-mediated myocardial fibrosis in DCM. Key follow-up experiments to address this include: 1) adoptive transfer of Treg cells with Treg-specific Areg knockout (Aregfl/fl-Foxp3Cre) to confirm the requirement of Treg-derived Areg for myocardial fibrosis *in vivo*; 2) *in vivo* systemic neutralization of Areg using specific monoclonal antibodies during the adoptive transfer process; 3) fibroblast-specific blockade of EGFR (the canonical receptor of Areg) to verify that Areg exerts pro-fibrotic effects by directly activating cardiac fibroblasts. Additionally, while we have identified key molecular players such as DBC1 and Areg, the comprehensive signaling pathways and interactions involving other immune cells in the diabetic heart require further elucidation. Furthermore, the study primarily focused on specific Treg subsets, thus broader Treg heterogeneity in DCM could be overlooked. Looking forward, future efforts should aim to translate these findings into clinical settings, investigating the therapeutic potential of targeting the DBC1-HIF-1α-PPAR-γ axis or ST2^hi^Areg^hi^ cells in DCM patients. Expanding the investigation to include human tissues will be essential for validating our findings. Additionally, understanding the crosstalk between Treg cells and other immune cells within the myocardial microenvironment could provide novel insights into the immunopathogenesis of DCM. The development of targeted therapies that harness the immunomodulatory properties of Treg cells represents a promising avenue for DCM treatment in future research.

**Figure 8:**
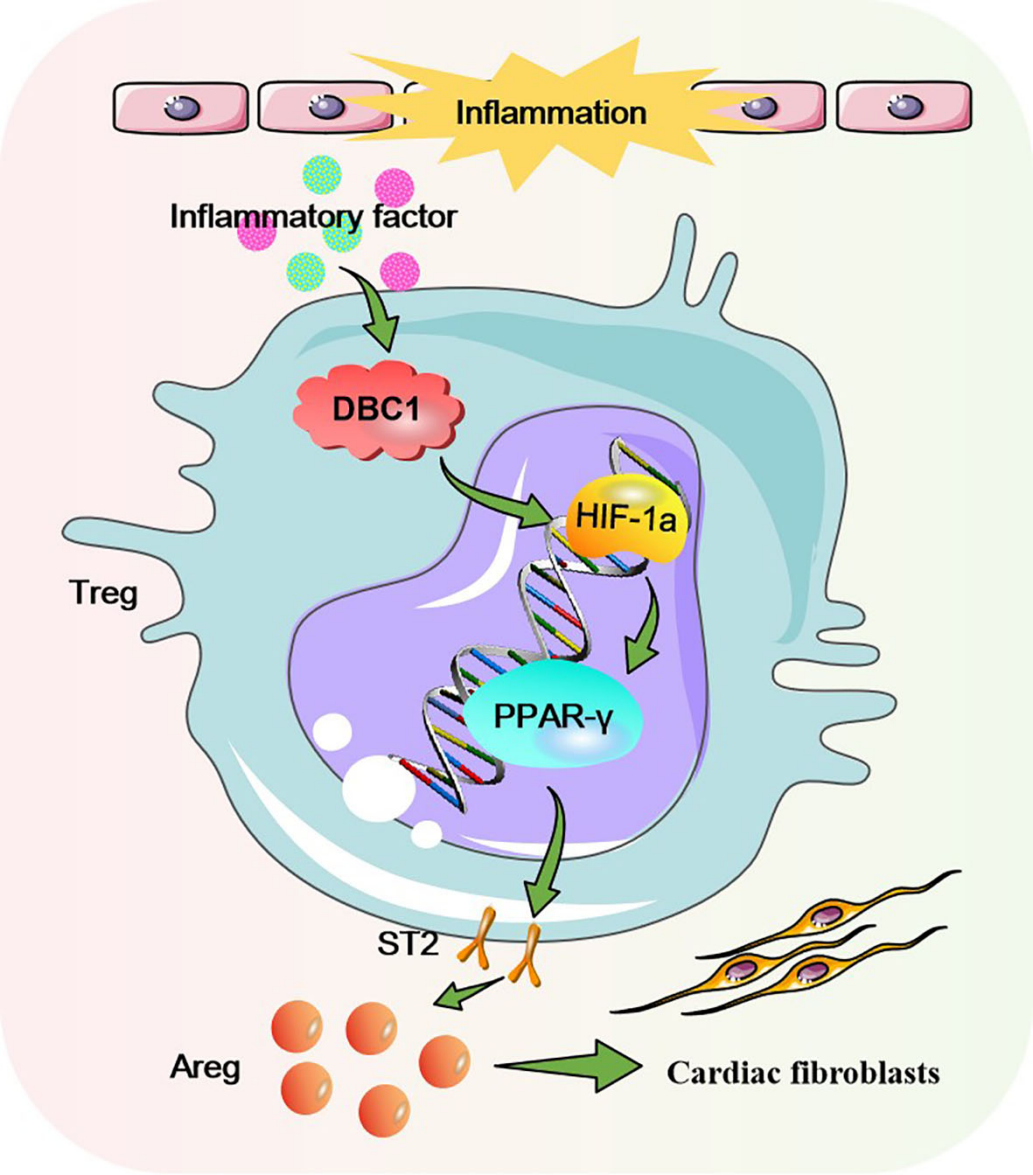
Schematic of the DBC1-HIF-1α-PPAR-γ axis in myocardial fibrosis of DCM. Under the chronic inflammatory condition of DCM, DBC1 activates the HIF-1α-PPAR-γ axis to induce the differentiation of cardiac Treg cells into the pro-fibrotic ST2^hi^Areg^hi^ subset, which drives pathological myocardial fibrosis and cardiac dysfunction through the paracrine secretion of Areg.

In summary, our current investigation indicates that ST2^hi^Areg^hi^ Treg subsets are increased in the hearts of DCM mice, and they enhance myocardial fibrosis by secreting Areg. The DBC1-HIF-1α-PPAR-γ axis is responsible for Treg cell differentiation into ST2^hi^Areg^hi^ subsets, as illustrated in **Figure 8**. Focusing on this novel signaling cascade could provide innovative therapeutic strategies for the treatment of DCM.

## Data Availability

The original contributions presented in the study are included in the article/**Supplementary Material**, further inquiries can be directed to the corresponding author.
