# Supplementary material for: The DBC1-HIF-1α-PPAR-γ axis regulates Treg cell differentiation to promote myocardial fibrosis in experimental diabetic cardiomyopathy through the paracrine secretion of Areg
Source: Front Endocrinol (Lausanne). 2026 Apr 13;17:1780666. doi: 10.3389/fendo.2026.1780666 (PMC13111003; doi:10.3389/fendo.2026.1780666)
Supplement: Supplementary file 1 [file DataSheet1.pdf]

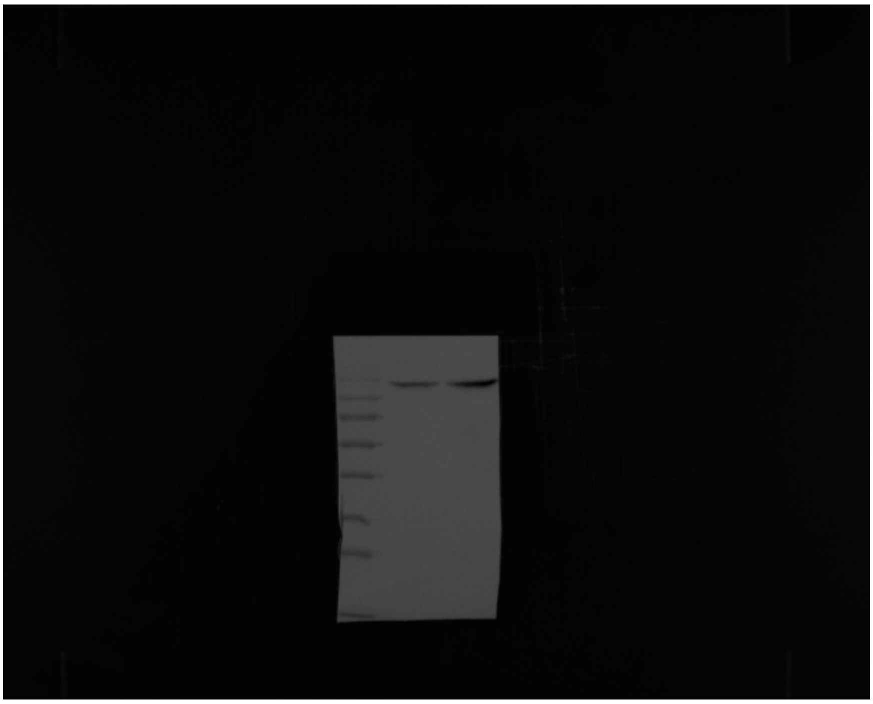

Figure 2-B (1-DBC1)

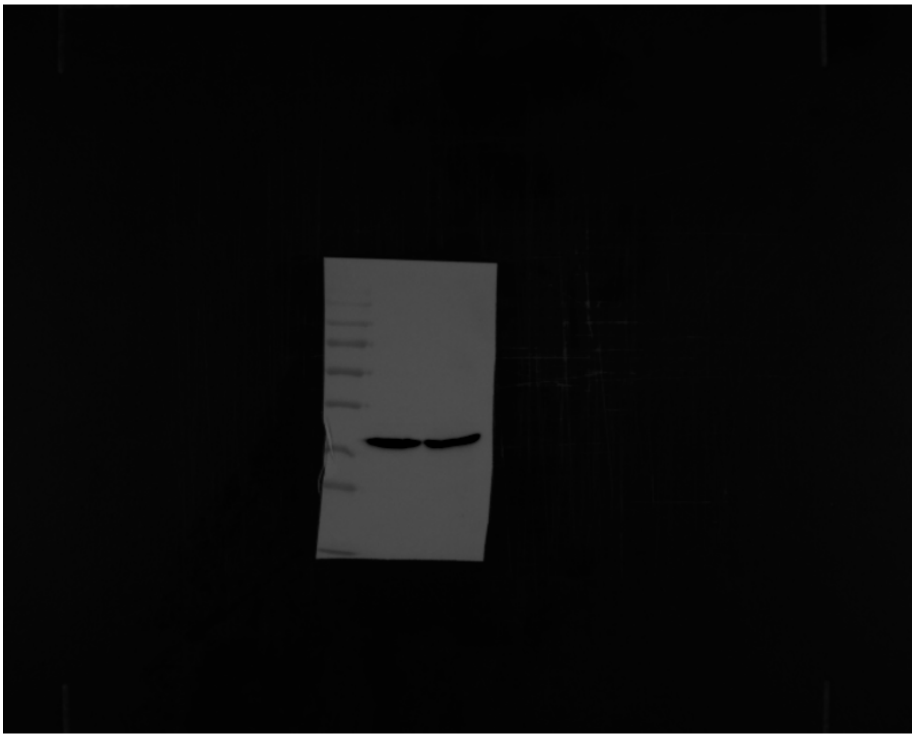

Figure 2-B (2-GAPDH)

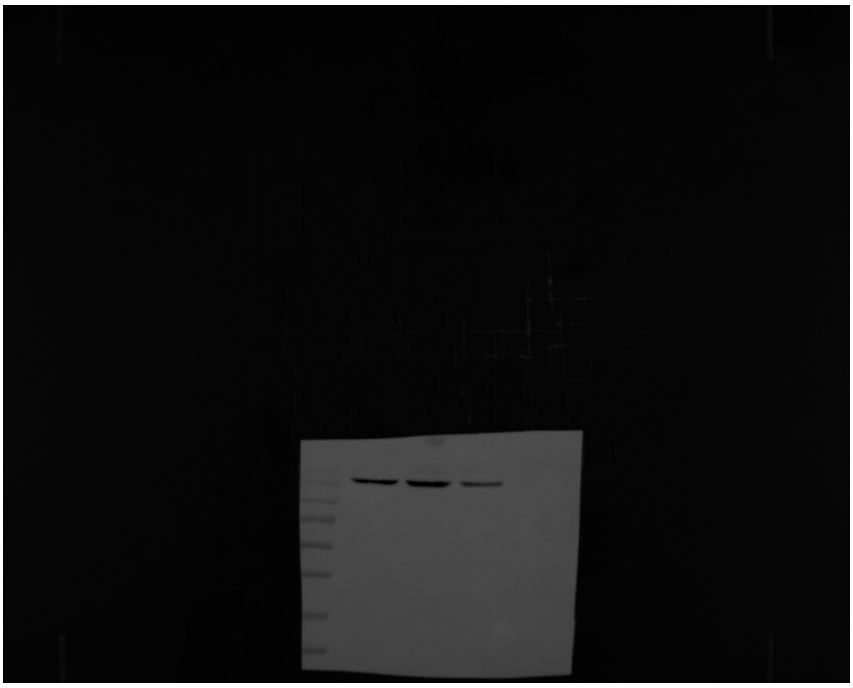

Figure 2-D (1-DBC1)

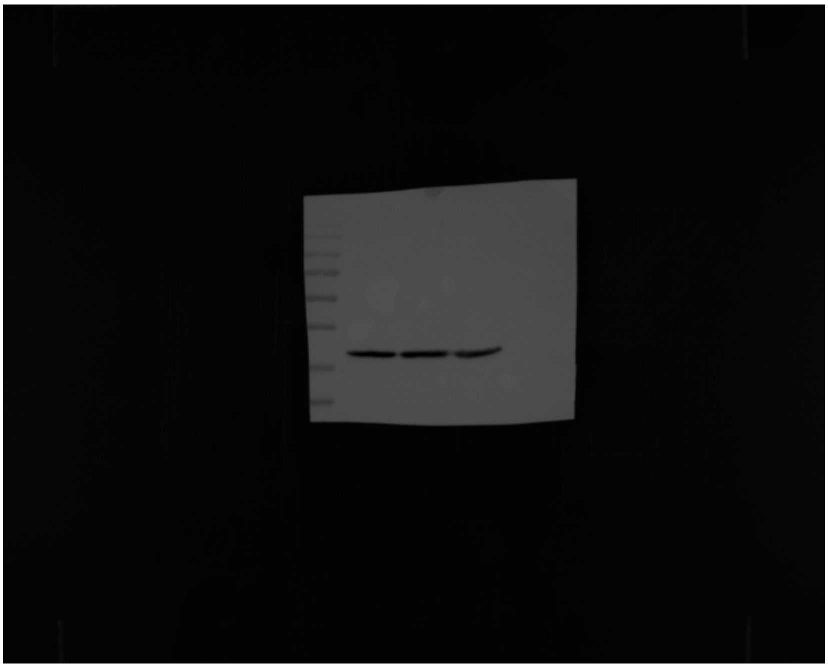

Figure 2-D (2-GAPDH)

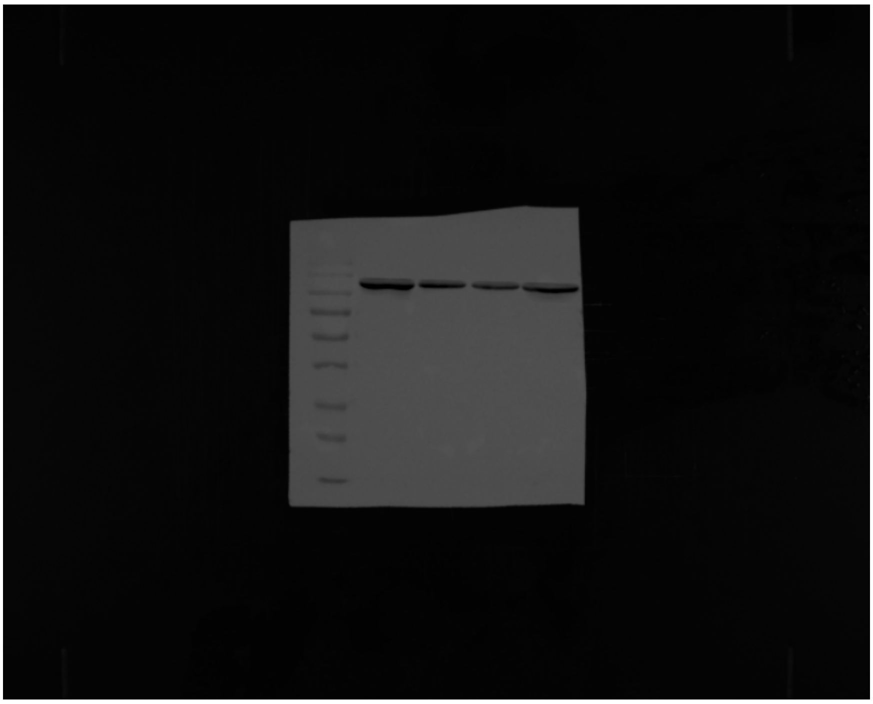

Figure 3-D (1-collagen i)

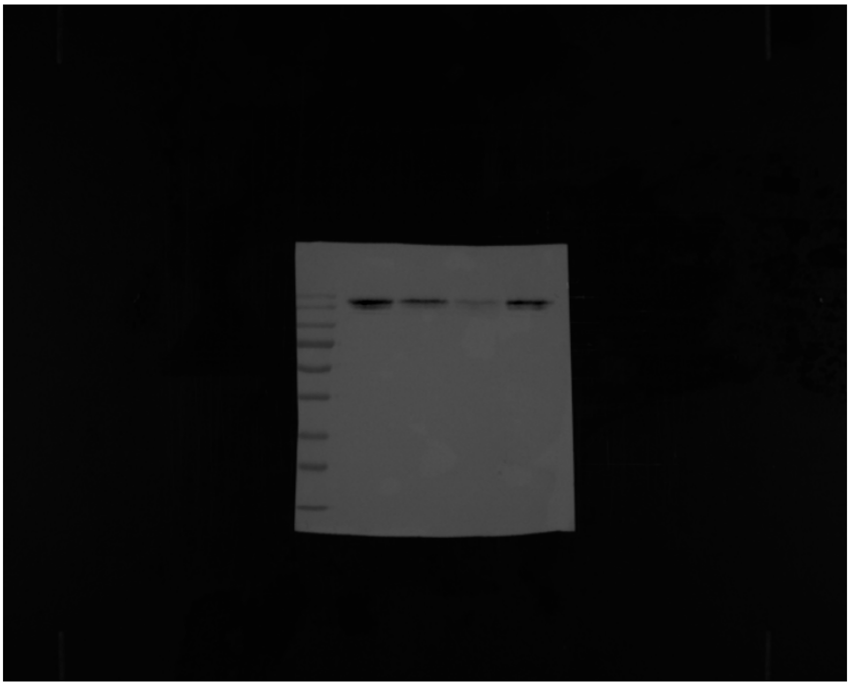

Figure 3-D (2-collagen iii)

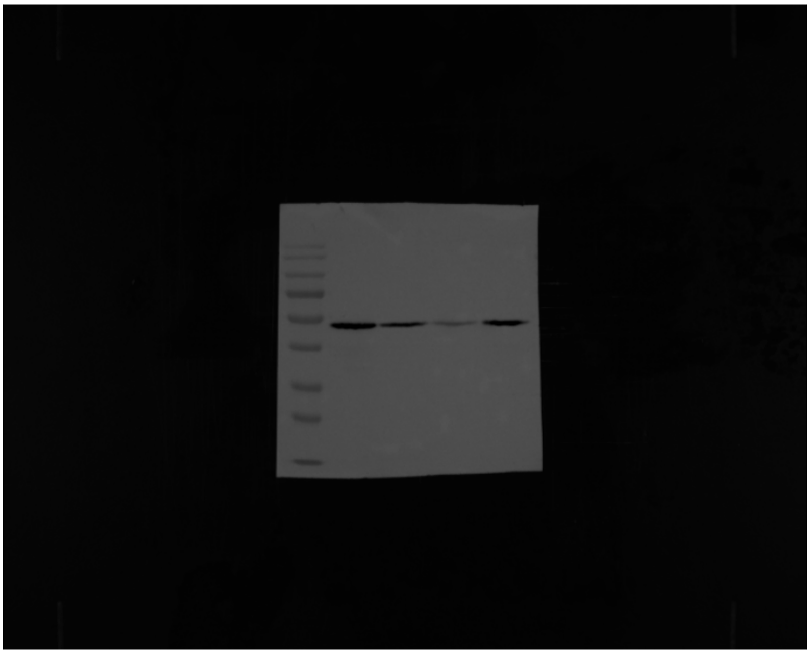

Figure 3-D (3-MMP-3)

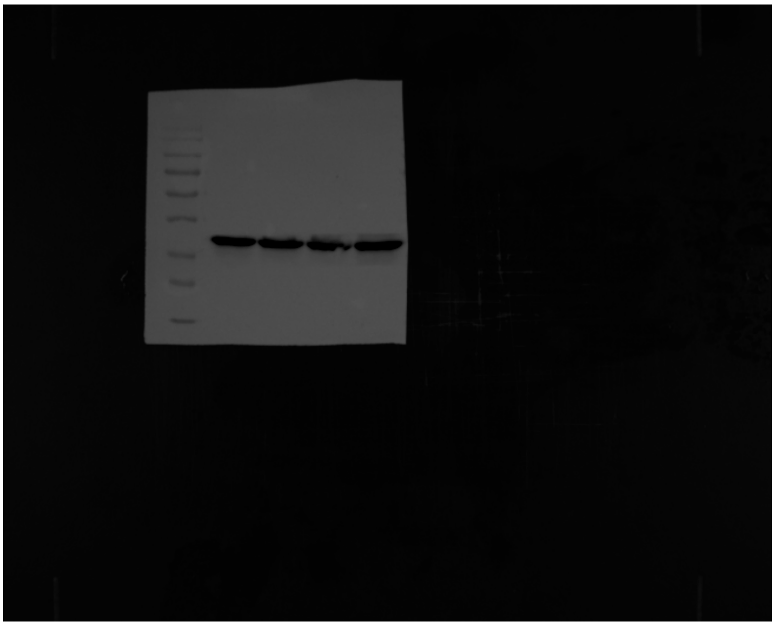

Figure 3-D (4-GAPDH)

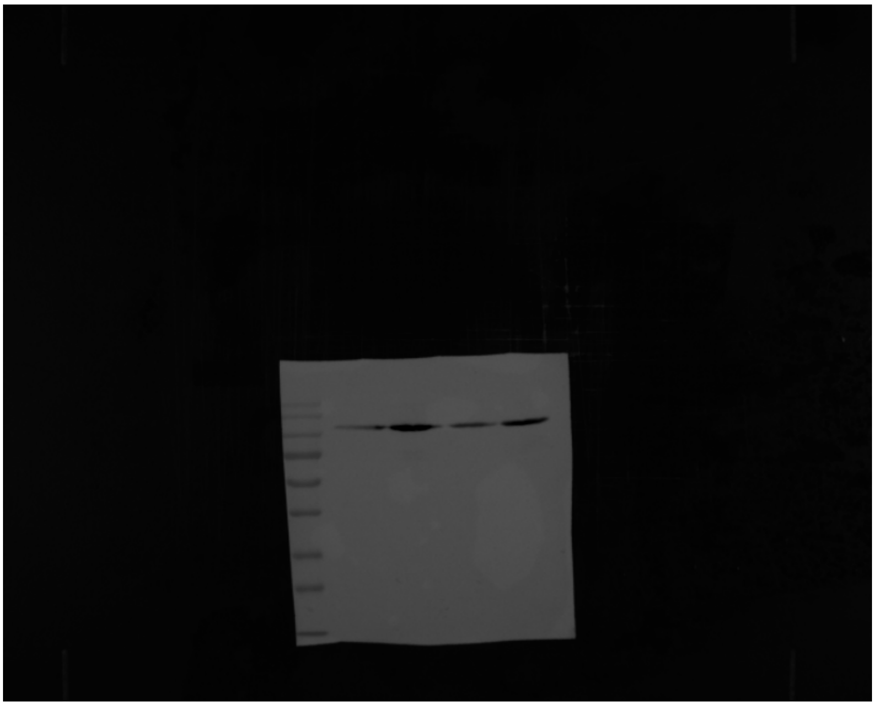

Figure 6-B (1-HIF-1α)

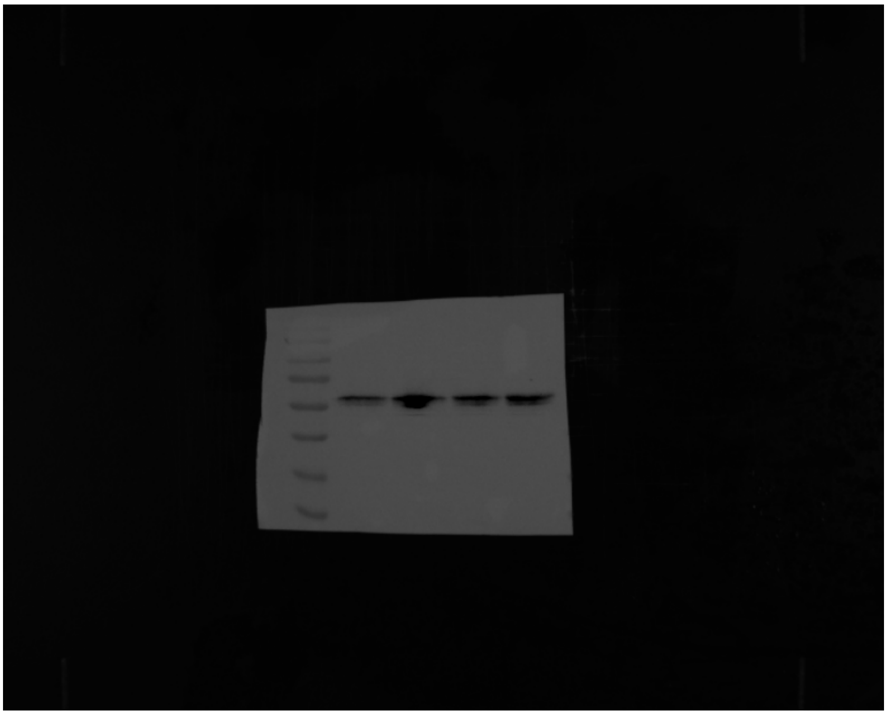

Figure 6-B (2-PPAR-γ)

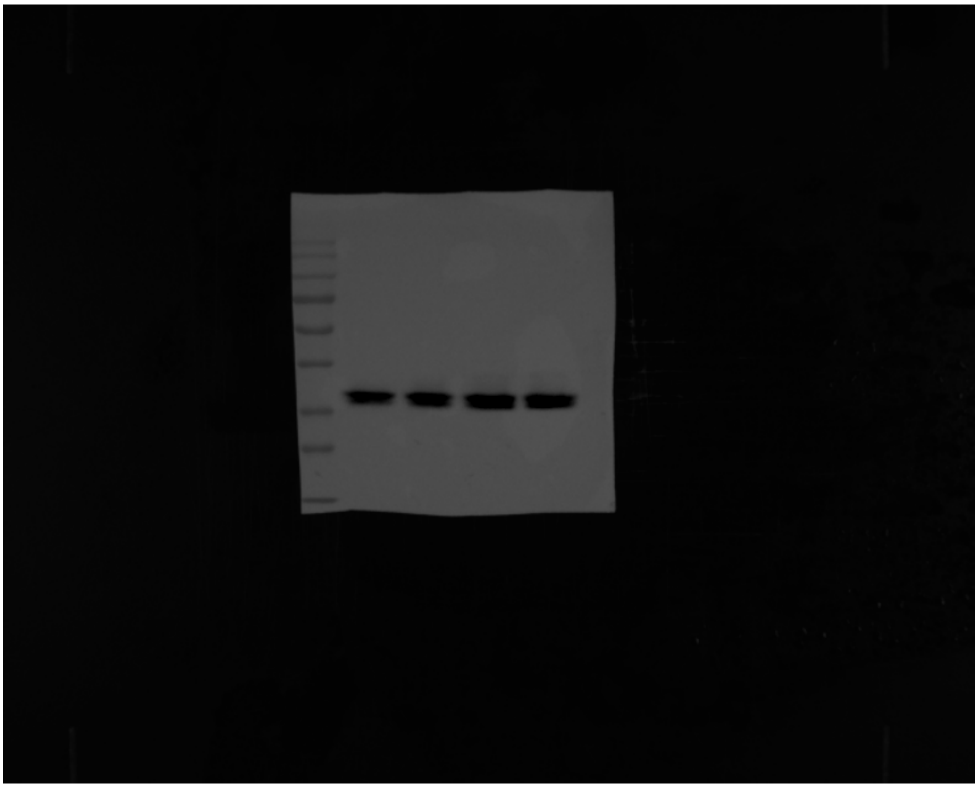

Figure 6-B (3-GAPDH)
