# Supplementary material for: The DBC1-HIF-1α-PPAR-γ axis regulates Treg cell differentiation to promote myocardial fibrosis in experimental diabetic cardiomyopathy through the paracrine secretion of Areg
Source: Front Endocrinol (Lausanne). 2026 Apr 13;17:1780666. doi: 10.3389/fendo.2026.1780666 (PMC13111003; doi:10.3389/fendo.2026.1780666)

Supplementary Material for

The DBC1-HIF-1α-PPAR-γ axis regulates Treg cell differentiation to promote myocardial fibrosis in experimental diabetic cardiomyopathy through the paracrine secretion of Areg

Fig. S1 Gating strategy of hepatic Foxp3+ Tregs.

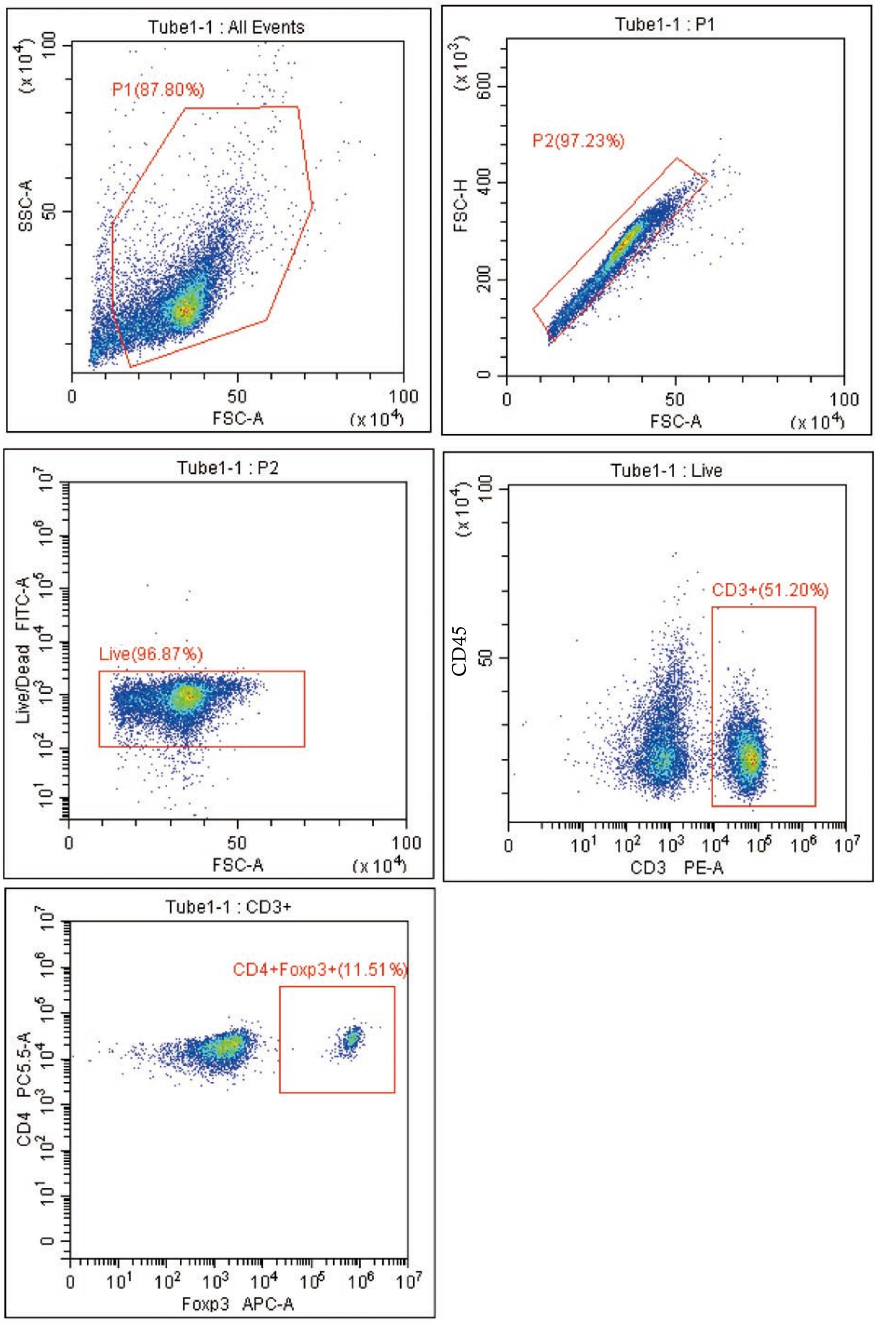

Fig. S2 Immunoblot assay confirmed the specific knockout of DBC1 in Treg cells.

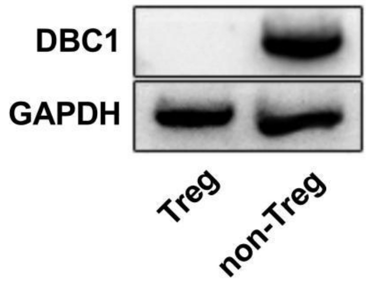

Supplement: Supplementary file 2 [file DataSheet2.pdf]
